# Supplementary material for: Regulation of cytokine signaling through direct interaction between cytokine receptors and the ATG16L1 WD40 domain
Source: Nat Commun. 2020 Nov 20;11:5919. doi: 10.1038/s41467-020-19670-4 (PMC7679444; doi:10.1038/s41467-020-19670-4)
Supplement: Supplementary file 1 — Supplementary Information [file 41467_2020_19670_MOESM1_ESM.pdf]

SUPPLEMENTARY FIGURES

a

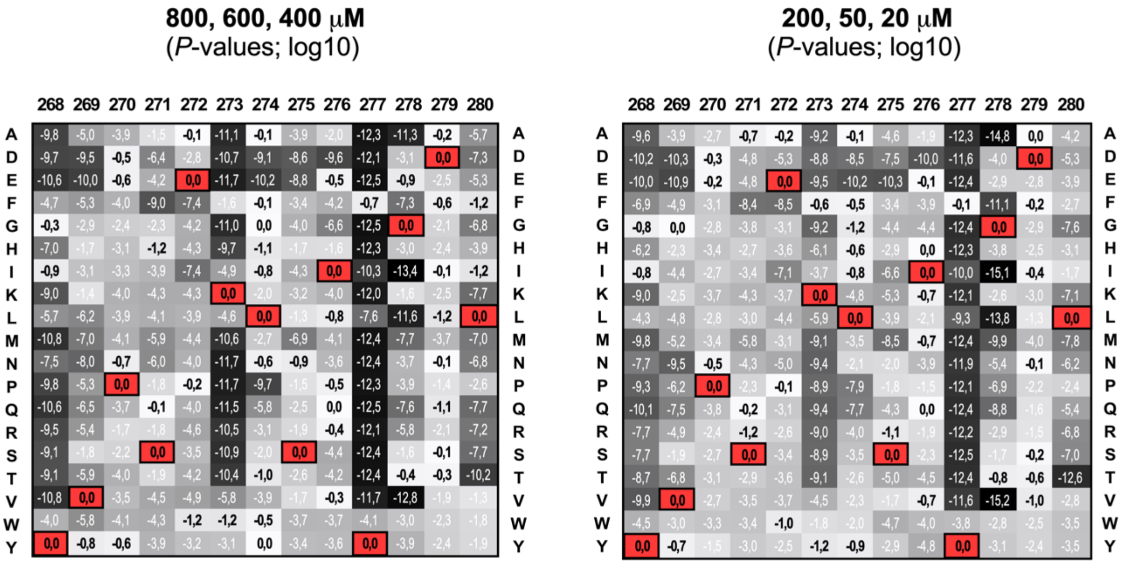

b

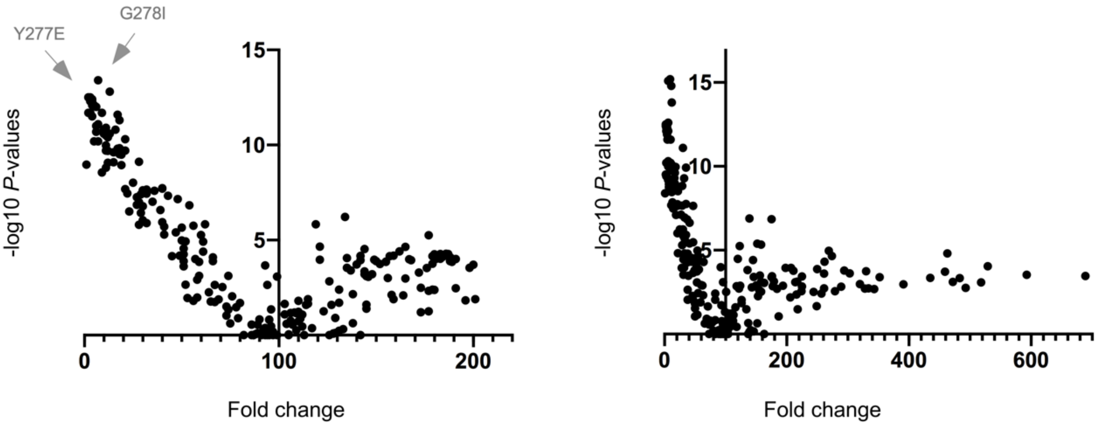

Supplementary Figure 1. Statistics of peptide microarray WDD binding studies. a, Heatmaps displaying log10 *P*-values of the differences in binding activity observed between a given mutant and the wild-type peptide (n=8 experimental points). Numbers below -1,3 (*P*-value>0,05; significant differences) are shown in white font; values above this threshold are not significant and are represented in black/bold font. Squares are coloured following a continuous grey scale from low (white) to high (black) significance (lower *P*-value). Squares in red represent the native aminoacids present at each position in the 268-280 TMEM59 peptide. **b,** Volcano plots representing fold changes in the binding activity of the mutant peptides against the statistical significance of the change (-log10 *P*-values). Examples of 2 substitutions showing particularly strong binding defects that are highly significant (Y277E and G278I) are indicated. Source data are provided as a Source Data file.

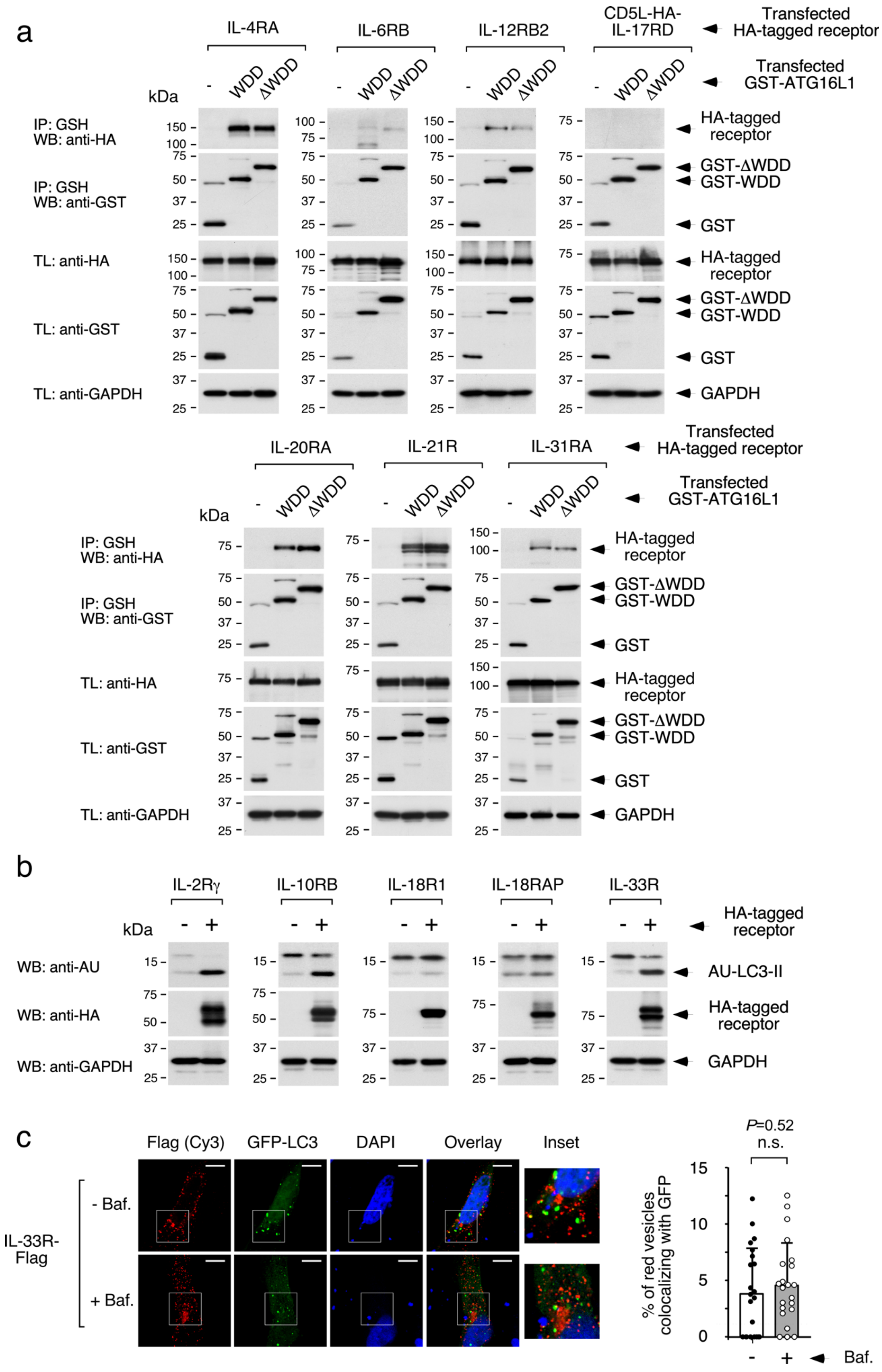
a
IL-4RA
IL-6RB
IL-12RB2
CD5L-HA-IL-17RD
Transfected HA-tagged receptor
Transfected GST-ATG16L1
- WDD ΔWDD
kDa
IP: GSH WB: anti-HA
IP: GSH WB: anti-GST
TL: anti-HA
TL: anti-GST
TL: anti-GAPDH
HA-tagged receptor
GST-ΔWDD
GST-WDD
GST
GAPDH
IL-20RA
IL-21R
IL-31RA
b
IL-2Rγ
IL-10RB
IL-18R1
IL-18RAP
IL-33R
WB: anti-AU
WB: anti-HA
WB: anti-GAPDH
AU-LC3-II
c
Flag (Cy3)
GFP-LC3
DAPI
Overlay
Inset
IL-33R-Flag
- Baf.
+ Baf.
P=0.52
n.s.
% of red vesicles colocalizing with GFP
Baf.

Supplementary Figure 2. Selection of candidate receptors. a, Co-immunoprecipitation studies to identify receptors able to specifically interact with the WDD. HEK-293T cells were co-transfected with tagged forms of the indicated cytokine receptors along with constructs including the WDD (320-607) or deltaWDD (1-319) fused to GST, as shown. Cells were lysed 36 h later and subjected to GST immunoprecipitation with agarose beads coupled to GSH. Shown are Western-blots against the indicated molecules. IP: immunoprecipitate; TL: total lysate. This panel shows candidate receptors unable to bind the WDD, or able to do so non-specifically due to additional interaction with deltaWDD. **b,** Evaluation of LC3 lipidation activity induced by the candidate cytokine receptors. HEK-293T cells were transfected with the indicated tagged cytokine receptors along with a plasmid expressing AU-LC3. Cells were lysed 36 h post-transfection for Western-blotting against the indicated molecules. **c,** Colocalization between transfected IL-33R and co-expressed GFP-LC3. HeLa cells were transfected with plasmids expressing IL-33R-Flag and GFP-LC3. Cells were treated for the last 6 h of culture with bafilomycin A1 (75 nM, as indicated), fixed 36 h post-transfection, stained with an anti-Flag antibody and scored for colocalization between the flag signal and GFP-LC3. Shown are representative confocal pictures (left), and a graph displaying the percentage of Flag-positive vesicles colocalizing with the GFP-positive signal -/+ s.d. (right; n=22 cells; n.s: $P>0,05$, two-sided Student's *t*-test). Source data are provided as a Source Data file.

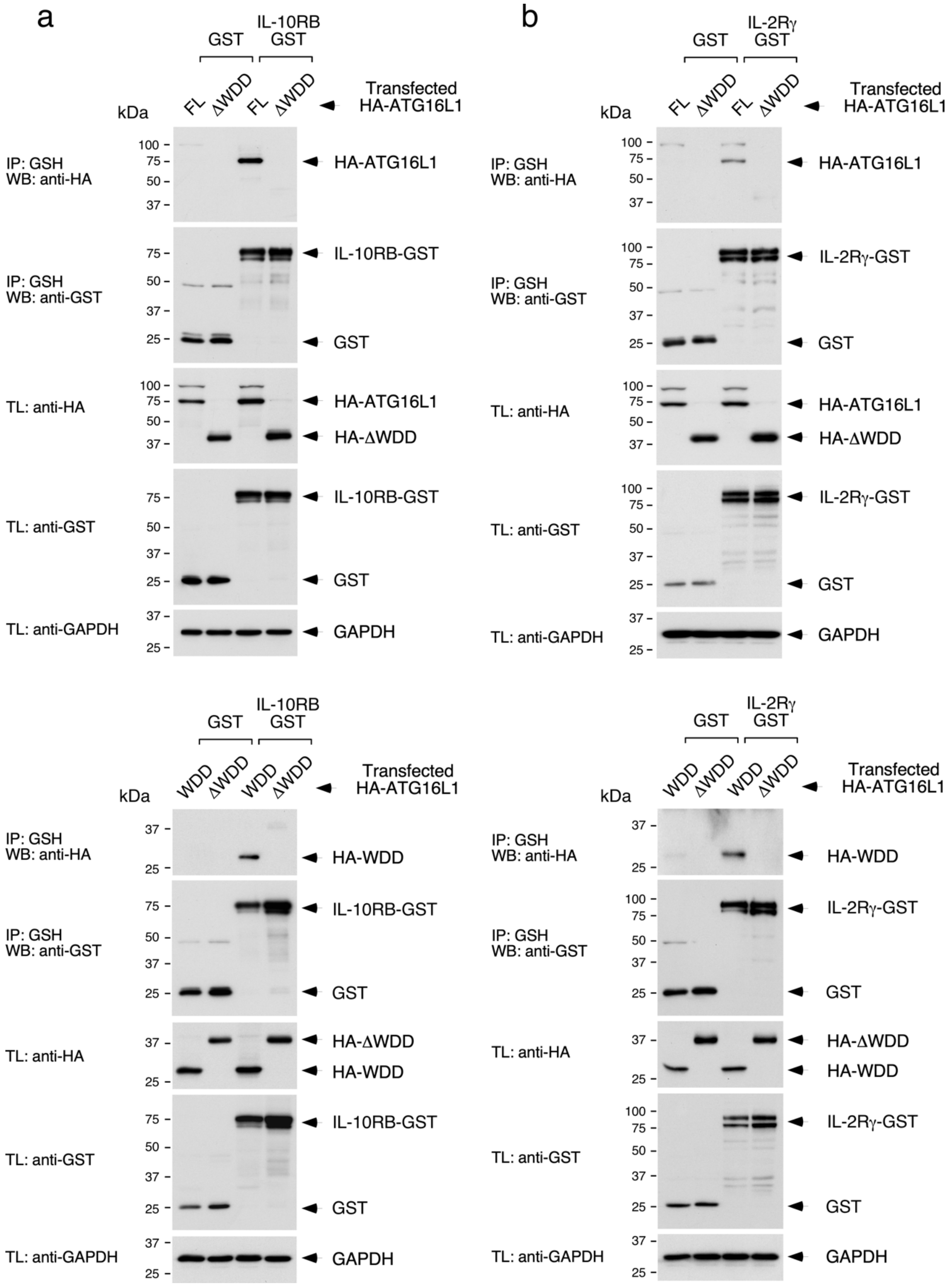
a
GST
IL-10RB GST
FL
ΔWDD
Transfected HA-ATG16L1
kDa
IP: GSH WB: anti-HA
IP: GSH WB: anti-GST
TL: anti-HA
TL: anti-GST
TL: anti-GAPDH
HA-ATG16L1
IL-10RB-GST
GST
HA-ΔWDD
GAPDH
WDD
HA-WDD
b
IL-2Rγ GST
IL-2Rγ-GST
100
75
50
37
25

Supplementary Figure 3. Co-immunoprecipitation studies between IL-10RB/IL-2Rg fused to GST and full-length or deleted HA-ATG16L1 constructs. HEK-293T cells were co-transfected with the indicated receptors (**a,** IL-10RB; **b,** IL-2Rg) fused to GST and the shown HA-tagged ATG16L1 constructs (WDD: 320-607; deltaWDD: 1-319). Cells were lysed 36 h later and subjected to GST immunoprecipitation with agarose beads coupled to GSH. Shown are Western-blots against the indicated molecules. IP: immunoprecipitate; TL: total lysate. Source data are provided as a Source Data file.

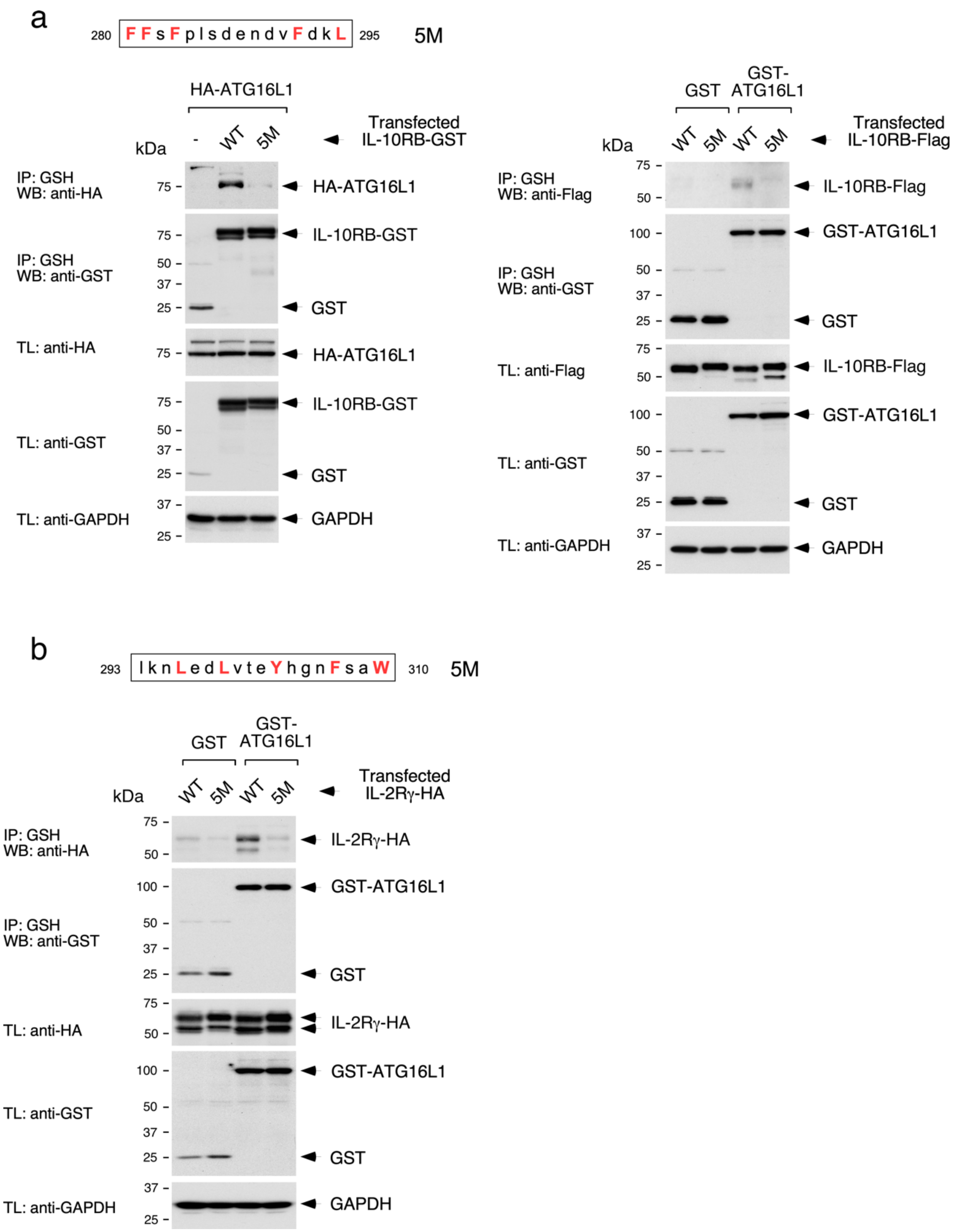
a
280 FFsFplsdendvFdkL 295
5M
HA-ATG16L1
kDa
-
WT
5M
Transfected IL-10RB-GST
IP: GSH WB: anti-HA
HA-ATG16L1
IL-10RB-GST
IP: GSH WB: anti-GST
GST
TL: anti-HA
HA-ATG16L1
IL-10RB-GST
TL: anti-GST
GST
TL: anti-GAPDH
GAPDH
GST
GST-ATG16L1
Transfected IL-10RB-Flag
IP: GSH WB: anti-Flag
IL-10RB-Flag
GST-ATG16L1
IP: GSH WB: anti-GST
GST
TL: anti-Flag
IL-10RB-Flag
GST-ATG16L1
TL: anti-GST
GST
TL: anti-GAPDH
GAPDH
b
293 lknLedLvteYhgnFsaW 310
5M
GST
GST-ATG16L1
Transfected IL-2Rγ-HA
IP: GSH WB: anti-HA
IL-2Rγ-HA
GST-ATG16L1
IP: GSH WB: anti-GST
GST
TL: anti-HA
IL-2Rγ-HA
GST-ATG16L1
TL: anti-GST
GST
TL: anti-GAPDH
GAPDH

Supplementary Figure 4. Co-immunoprecipitation studies between wild-type or motif-mutated versions of IL-10RB/IL-2Rg and full-length ATG16L1. HEK-293T cells were co-transfected with wild-type (WT) or motif-mutated (5M, residues in red font mutated to alanine) forms of IL-10RB (**a**) or IL-2Rg (**b**) along with the indicated constructs expressing ATG16L1. Cells were lysed 36 h later and subjected to GST immunoprecipitation with agarose beads coupled to GSH. Shown are Western-blots against the indicated molecules. IP: immunoprecipitate; TL: total lysate. Source data are provided as a Source Data file.

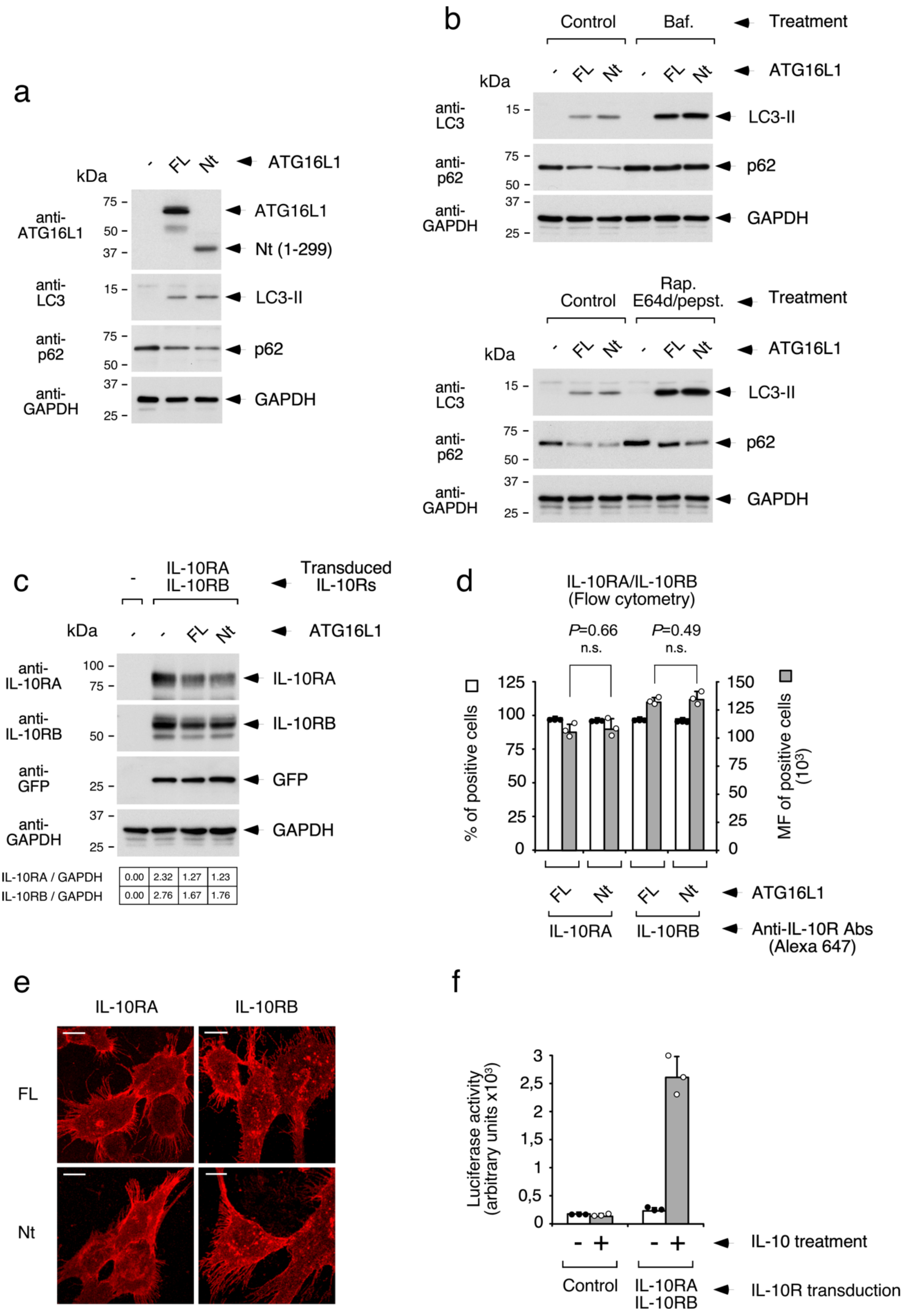
a
kDa
ATG16L1
FL
Nt
anti-ATG16L1
anti-LC3
anti-p62
anti-GAPDH
75
50
37
15
25
ATG16L1
Nt (1-299)
LC3-II
p62
GAPDH
b
Control
Baf.
Rap. E64d/pepst.
Treatment
ATG16L1
LC3-II
p62
GAPDH
c
-
IL-10RA IL-10RB
Transduced IL-10Rs
anti-IL-10RA
anti-IL-10RB
anti-GFP
100
IL-10RA
IL-10RB
GFP
IL-10RA / GAPDH 0.00 2.32 1.27 1.23
IL-10RB / GAPDH 0.00 2.76 1.67 1.76
d
IL-10RA/IL-10RB (Flow cytometry)
P=0.66 n.s.
P=0.49 n.s.
% of positive cells
MF of positive cells (10³)
Anti-IL-10R Abs (Alexa 647)
e
FL
Nt
f
Luciferase activity (arbitrary units x10³)
IL-10 treatment
Control
IL-10R transduction

Supplementary Figure 5. Characterization of *Atg16L1-/-* MEFs engineered to test the role of the WDD in IL-10 signalling. *Atg16L1-/-* MEFs harbouring the STAT3-luciferase reporter and expressing STAT3 were restored with full-length ATG16L1 (FL) or a deleted version lacking the WDD (Nt, 1-299), and retrovirally transduced to express IL-10Rs A and B. **a,** Expression levels of full-length ATG16L1 (FL) and the N-terminal domain (1-299; Nt) in the engineered cells. The indicated cells were lysed for Western-blot against the shown molecules. **b,** Evaluation of autophagic flux in *Atg16L1-/-* MEFs restored with FL or Nt. Cells were treated with bafilomycin A1 (75 nM, 6 h; top panel) or rapamycin (2 uM) plus E64d/pepstatin (10 ug/ml each; bottom panel) for 10 h, and lysed for Western-blotting with the shown antibodies. **c,** Expression levels of IL-10RA and IL-10RB in the engineered MEFs. Cells were lysed for Western-blot against the shown molecules. The anti-GFP blot detects GFP expression from an IRES-GFP cassette located downstream the IL-10RB open reading frame in the retroviral vector. Equal GFP expression levels argue that viral transduction was comparable in all cases, indicating that the increased IL-10R expression in *Atg16L1-/-* cells is not due to enhanced transduction efficiency. Corrected densitometric quantifications are shown at the bottom of the Western-blot images. **d,** Surface expression levels of IL-10Rs. Cells were subjected to flow cytometry with specific anti-IL-10RA and -IL-10RB antibodies. Shown are the average percentage of positive cells with respect to control cells lacking IL-10Rs (left axis, white bars, solid dots) and the average mean of fluorescence of the positive cells (MF, right axis, grey bars, white dots), from triplicate experimental points -/+ s.d. (n=3; n.s. *P*-value>0,05, two-sided Student's *t*-test)). A scheme of the gating and quantification strategy is provided in Supplementary Figure 17. **e,** Surface expression levels of IL-10Rs measured by immunofluorescence. The indicated unpermeabilized cells were stained *in vivo* with the same directly-conjugated anti-IL-10R antibodies as in **d** and fixed for immunofluorescence. Shown are representative confocal pictures. **f,** STAT3-luciferase response activated by IL-10 in *Atg16L1-/-* MEFs restored with full-length ATG16L1 and lacking or expressing IL-10Rs. The indicated cells were treated with IL-10 (50 ng/ml; 8 h) and lysed to measure luciferase activity. Show are mean values -/+ s.d. of a representative experiment carried out in triplicate experimental points (n=3). Source data are provided as a Source Data file.

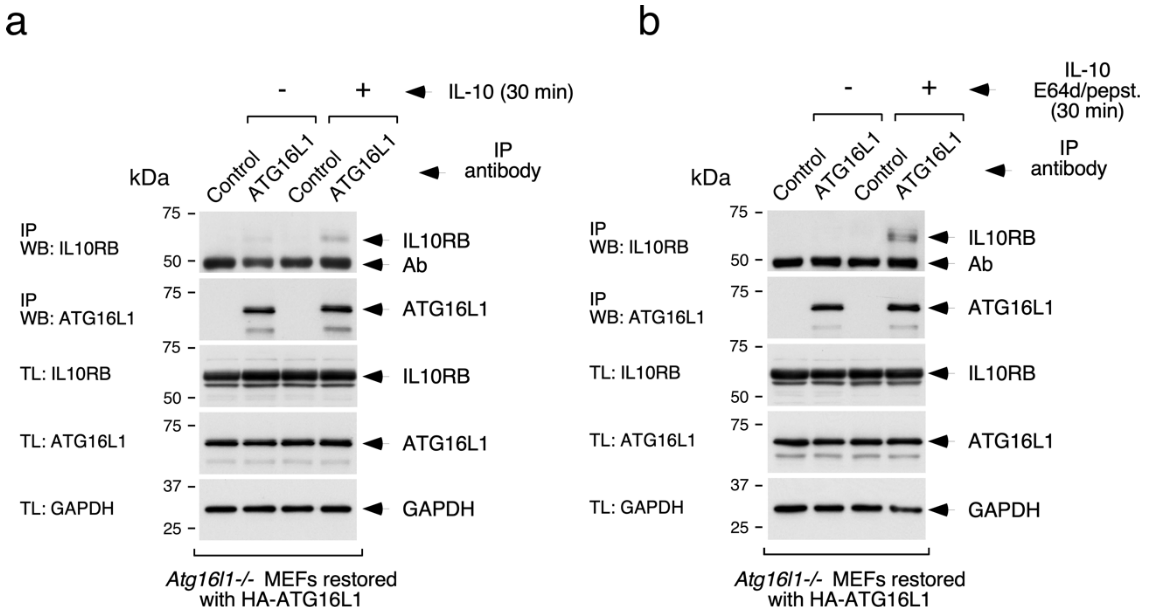
a
-
+
IL-10 (30 min)
Control
ATG16L1
Control
ATG16L1
IP antibody
kDa
IP WB: IL10RB
WB: ATG16L1
TL: IL10RB
TL: ATG16L1
TL: GAPDH
75
50
37
25
IL10RB
Ab
ATG16L1
GAPDH
Atg16l1-/- MEFs restored with HA-ATG16L1
b
IL-10 E64d/pepst. (30 min)
Control
ATG16L1
Control
ATG16L1
IP antibody
kDa
IP WB: IL10RB
IP WB: ATG16L1
TL: IL10RB
TL: ATG16L1
TL: GAPDH
IL10RB
Ab
ATG16L1
GAPDH
Atg16l1-/- MEFs restored with HA-ATG16L1

Supplementary Figure 6. IL-10-induced interaction between ATG16L1 and IL-10RB. *Atg16L1*-/- MEFs restored with full-length ATG16L1, and engineered to express IL-10Rs (the same cell line characterized in Supplementary Fig. 5), were treated with IL-10 (50 ng/ml; 30 min; panel **a**) or with IL-10 (50 ng/ml) and E64d/pepstatin (10 ug/ml each) for 30 min (panel **b**). Cells were lysed for immunoprecipitation with control (irrelevant rabbit Ig) or anti-ATG16L1 antibodies (as displayed). Shown are Western-blots against the indicated molecules. IP: immunoprecipitate; TL: total lysate. Ab refers to for immunoprecipitating antibody signal. Source data are provided as a Source Data file.

a

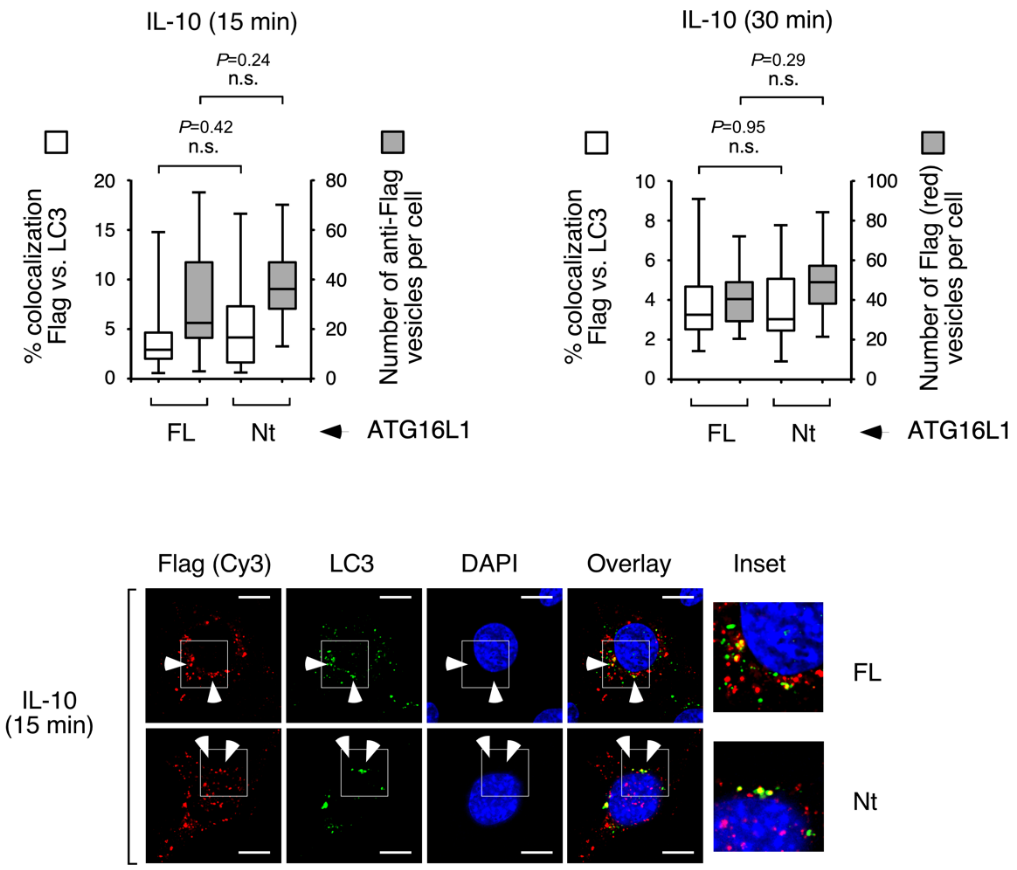
IL-10 (15 min)
P=0.24
n.s.
P=0.42
n.s.
% colocalization Flag vs. LC3
Number of anti-Flag vesicles per cell
FL
Nt
ATG16L1
IL-10 (30 min)
P=0.29
n.s.
P=0.95
n.s.
% colocalization Flag vs. LC3
Number of Flag (red) vesicles per cell
FL
Nt
ATG16L1
Flag (Cy3)
LC3
DAPI
Overlay
Inset
IL-10 (15 min)
FL
Nt

b

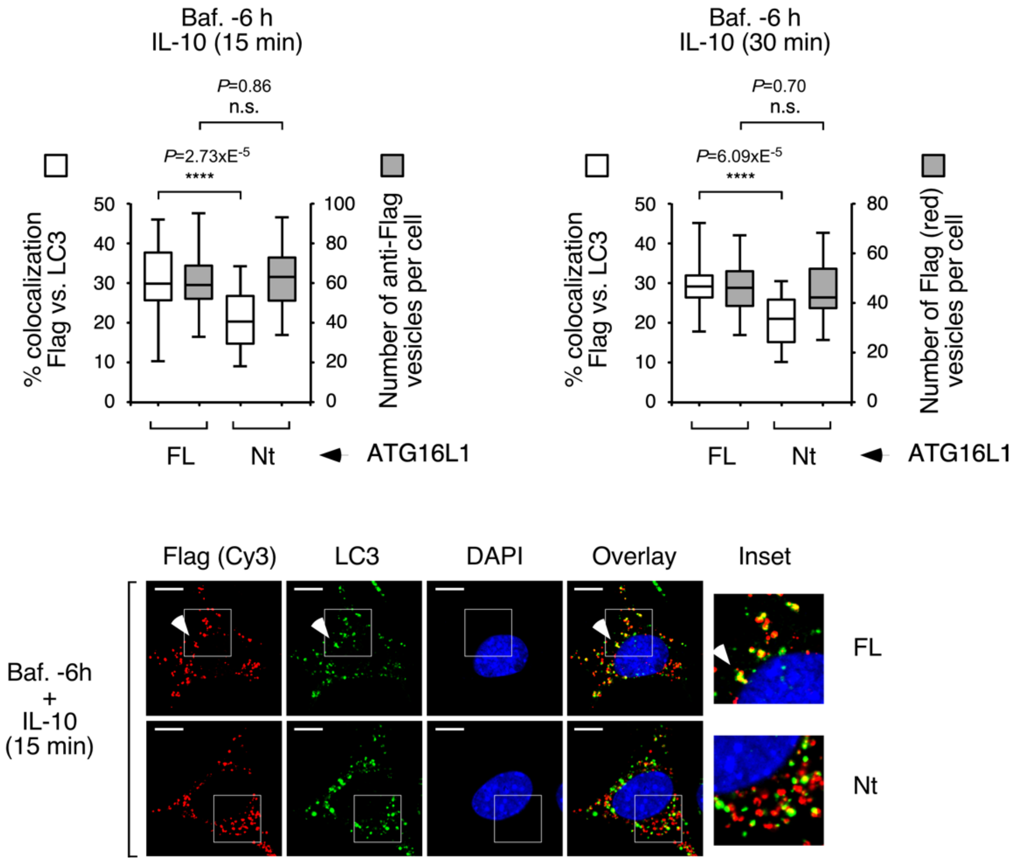
Baf. -6 h
IL-10 (15 min)
P=0.86
n.s.
P=2.73xE-5
****
% colocalization Flag vs. LC3
Number of anti-Flag vesicles per cell
FL
Nt
ATG16L1
Baf. -6 h
IL-10 (30 min)
P=0.70
n.s.
P=6.09xE-5
****
% colocalization Flag vs. LC3
Number of Flag (red) vesicles per cell
FL
Nt
ATG16L1
Flag (Cy3)
LC3
DAPI
Overlay
Inset
Baf. -6h
+
IL-10
(15 min)
FL
Nt

Supplementary Figure 7. Immunofluorescence assays to evaluate colocalization between IL-10RB and LC3 upon IL-10 treatment. The indicated engineered MEFs characterized in Supplementary Fig. 5 (FL: FL ATG16L1; N: Nt domain, 1-299), and expressing ectopic LC3 via retroviral transduction, were treated with IL-10 (50 ng/ml) for the shown times without **(a)** or with **(b)** a 6 h pre-incubation period with bafilomycin A1 (100 nM). Cells were then fixed for immunofluorescence with anti-Flag (IL-10RB, red) and anti-LC3 (green) antibodies. Shown are representative confocal pictures of the 15 min time points. Arrows indicate examples of colocalization events. Graphs show the percentage of colocalizing red (Flag) and green (LC3) signals per cell (left axis, white bars), and the total number of Flag-positive vesicles per cell (right axis, grey bars) under the different conditions. Data are presented as box-plots where the central line represents the median value, the box shows percentiles 25-75 and the whiskers include the most extreme values (n=20 cells except for colocalization in 15 min IL-10 where n=25, and -6 h Baf. + 15 min IL-10, where n=27 cells; n.s: $P>0,05$; (****): $P<0.0001$, two-sided Student's *t*-test). Source data are provided as a Source Data file.

a

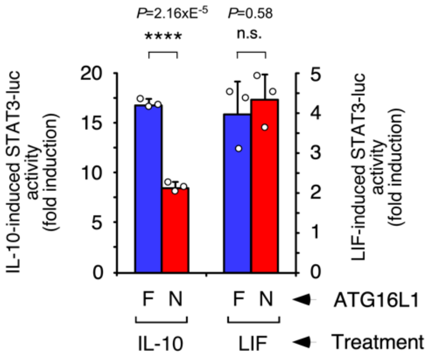

b

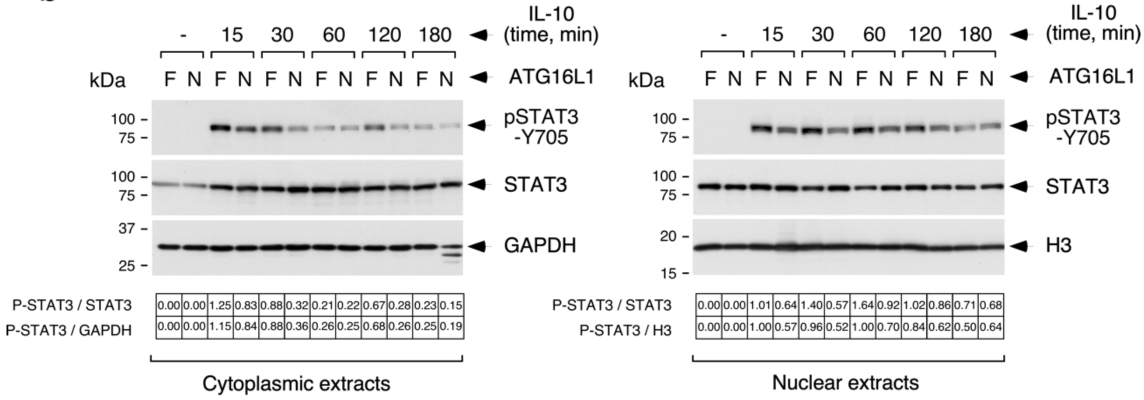

Cytoplasmic extracts

P-STAT3 / STAT3	0.00	0.00	1.25	0.83	0.88	0.32	0.21	0.22	0.67	0.28	0.23	0.15
P-STAT3 / GAPDH	0.00	0.00	1.15	0.84	0.88	0.36	0.26	0.25	0.68	0.26	0.25	0.19

Nuclear extracts

P-STAT3 / STAT3	0.00	0.00	1.01	0.64	1.40	0.57	1.64	0.92	1.02	0.86	0.71	0.68
P-STAT3 / H3	0.00	0.00	1.00	0.57	0.96	0.52	1.00	0.70	0.84	0.62	0.50	0.64

Supplementary Figure 8. a, Comparison of STAT3-luciferase activity induced by IL-10 and LIF in restored *Atg16L1*-/- MEFs engineered to express IL-10Rs. The same cell lines characterized in Supplementary Fig. 5 (F: FL ATG16L1; N: Nt domain, 1-299) were treated with IL-10 (50 ng/ml; 4 h; left axis) or LIF (50 ng/ml; 2 h; right axis), and lysed to measure luciferase activity. Data are expressed as the mean of fold induction of luciferase activity -/+ s.d obtained from triplicate experimental points (n=3; n.s.: $P>0.05$; (****): $P<0.0001$, two-sided Student's *t*-test) from a representative experiment. Blue bars: FL; Red bars: Nt. **b, IL-10-induced STAT3 phosphorylation.** Cells (F: FL ATG16L1; N: Nt domain) were continuously treated with IL-10 (50 ng/ml) for the shown times and lysed to obtain cytoplasmic and nuclear extracts for Western-blotting against the indicated molecules. Corrected densitometric quantifications are shown at the bottom of the Western-blot images. Source data are provided as a Source Data file.

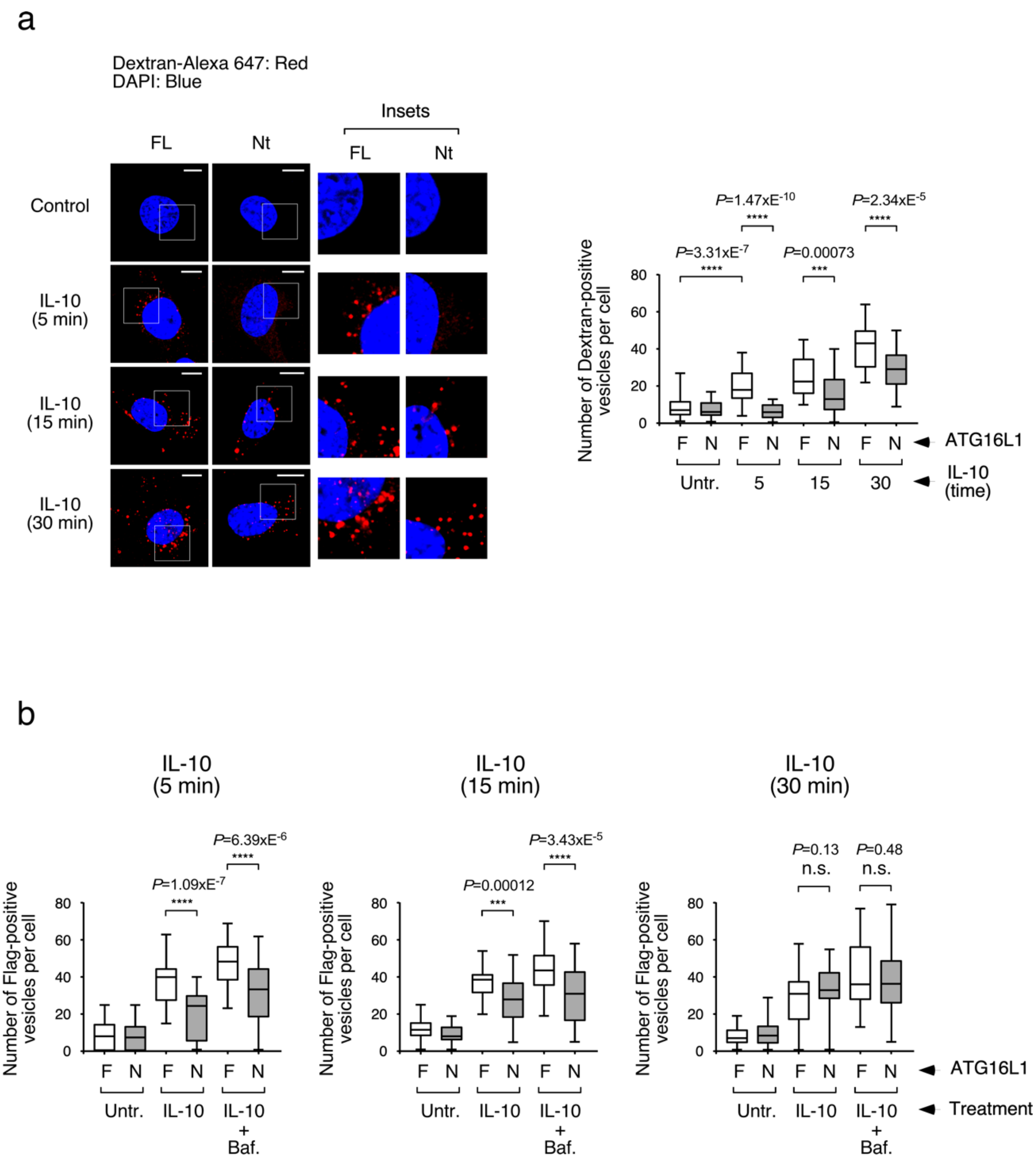
a
Dextran-Alexa 647: Red
DAPI: Blue
Insets
FL
Nt
FL
Nt
Control
IL-10
(5 min)
IL-10
(15 min)
IL-10
(30 min)
Number of Dextran-positive vesicles per cell
P=3.31xE-7
****
P=1.47xE-10
****
P=0.00073
***
P=2.34xE-5
****
0
20
40
60
80
F N F N F N F N
ATG16L1
Untr. 5 15 30
IL-10 (time)
b
IL-10 (5 min)
IL-10 (15 min)
IL-10 (30 min)
Number of Flag-positive vesicles per cell
P=1.09xE-7
****
P=6.39xE-6
****
P=0.00012
***
P=3.43xE-5
****
P=0.13
n.s.
P=0.48
n.s.
0
20
40
60
80
F N F N F N
Untr.
IL-10
IL-10 + Baf.
ATG16L1
Treatment

Supplementary Figure 9. a, Analysis of IL-10-induced endocytosis in cells expressing FL or Nt forms of ATG16L1 using dextran as an endocytic tracker. The indicated cells (FL or Nt) were co-incubated with IL-10 (50 ng/ml) and dextran-Alexa-647 (100 ug/ml) for the indicated times and then fixed and processed for immunofluorescence. Shown are representative confocal pictures (left). The numbers of dextran-positive vesicles per cell in the different conditions are presented as box-plots (right) where the central line represents the median value, the box shows percentiles 25-75 and the whiskers include the most extreme values (n=30 cells; (***): $P<0.001$; (****): $P<0.0001$, two-sided Student's *t*-test). **b, Pulse-chase experiment to evaluate the effect of bafilomycin in the number of intracellular IL-10RB-flag-positive vesicles induced by treatment with IL-10.** The indicated cell lines (FL or Nt) were pre-incubated with IL-10 (50 ng/ml; 30 min on ice) in the absence or presence of bafilomycin (75 nM, as indicated), washed to remove IL-10 and then chased for the indicated times (in the absence or presence of bafilomycin, as required) before fixation and staining for anti-Flag immunofluorescence. Box-plots display the number of Flag-positive vesicles per cell in the different conditions (the central line represents the median value, the box shows percentiles 25-75 and the whiskers include the most extreme values; n=40 cells except for untreated samples where n=20; n.s.: $P>0.05$; (***): $P<0.001$; (****): $P<0.0001$, two-sided Student's *t*-test). Source data are provided as a Source Data file.

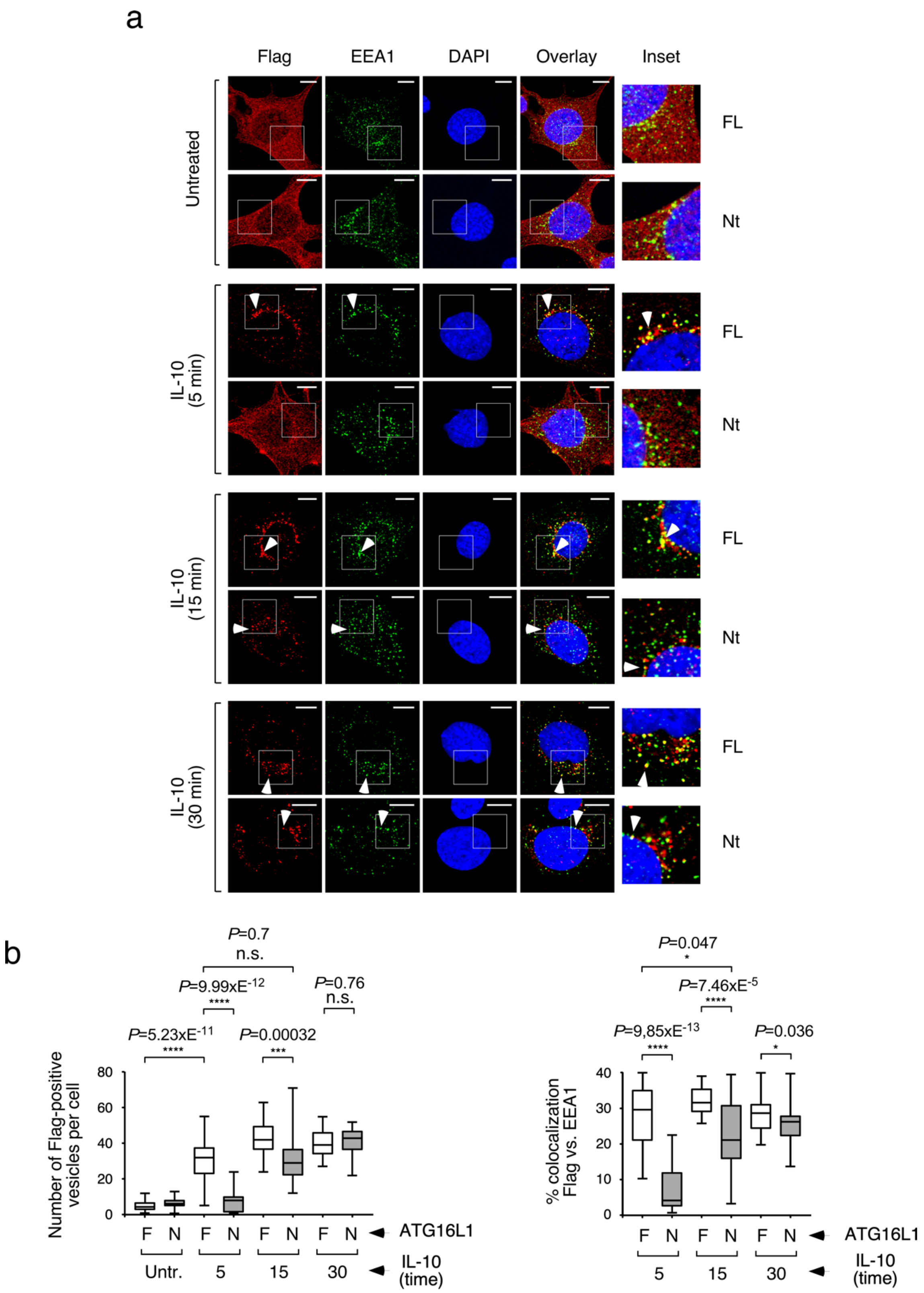
a
Flag
EEA1
DAPI
Overlay
Inset
Untreated
IL-10 (5 min)
IL-10 (15 min)
IL-10 (30 min)
FL
Nt
b
P=0.7 n.s.
P=9.99xE-12 ****
P=0.76 n.s.
P=5.23xE-11 ****
P=0.00032 ***
Number of Flag-positive vesicles per cell
0
20
40
60
80
F N F N F N F N
ATG16L1
Untr. 5 15 30
IL-10 (time)
P=0.047 *
P=7.46xE-5 ****
P=9,85xE-13 ****
P=0.036 *
% colocalization Flag vs. EEA1
0
10
20
30
40
F N F N F N
ATG16L1
5 15 30
IL-10 (time)

Supplementary Figure 10. Immunofluorescence pulse-chase assay to explore IL-10-induced IL-10RB endocytosis and intracellular trafficking to EEA1-positive compartments. a, The indicated cells (FL or Nt) were pre-incubated with IL-10 (50 ng/ml; 30 min on ice), washed to remove IL-10 and then chased for the indicated times before fixation and staining with anti-Flag (IL-10RB, red) and anti-EEA1 (green) antibodies. Shown are representative confocal pictures. Arrows indicate examples of colocalization events. **b,** Box-plots displaying quantification of the phenotypes observed in **a**. The left panel shows the number of Flag-positive vesicles per cell at different times after IL-10 treatment observed in this experiment. The right panel represents the percentage of colocalizing red (Flag) and green (EEA1) signals per cell. Data are presented as box-plots where the central line represents the median value, the box shows percentiles 25-75 and the whiskers include the most extreme values. For both panels, n=25 cells except for untreated cells (n=15); (n.s: P>0,05; (*): P<0.05; (***): P<0.001; (****): P<0.0001, two-sided Student's *t*-test). Source data are provided as a Source Data file.

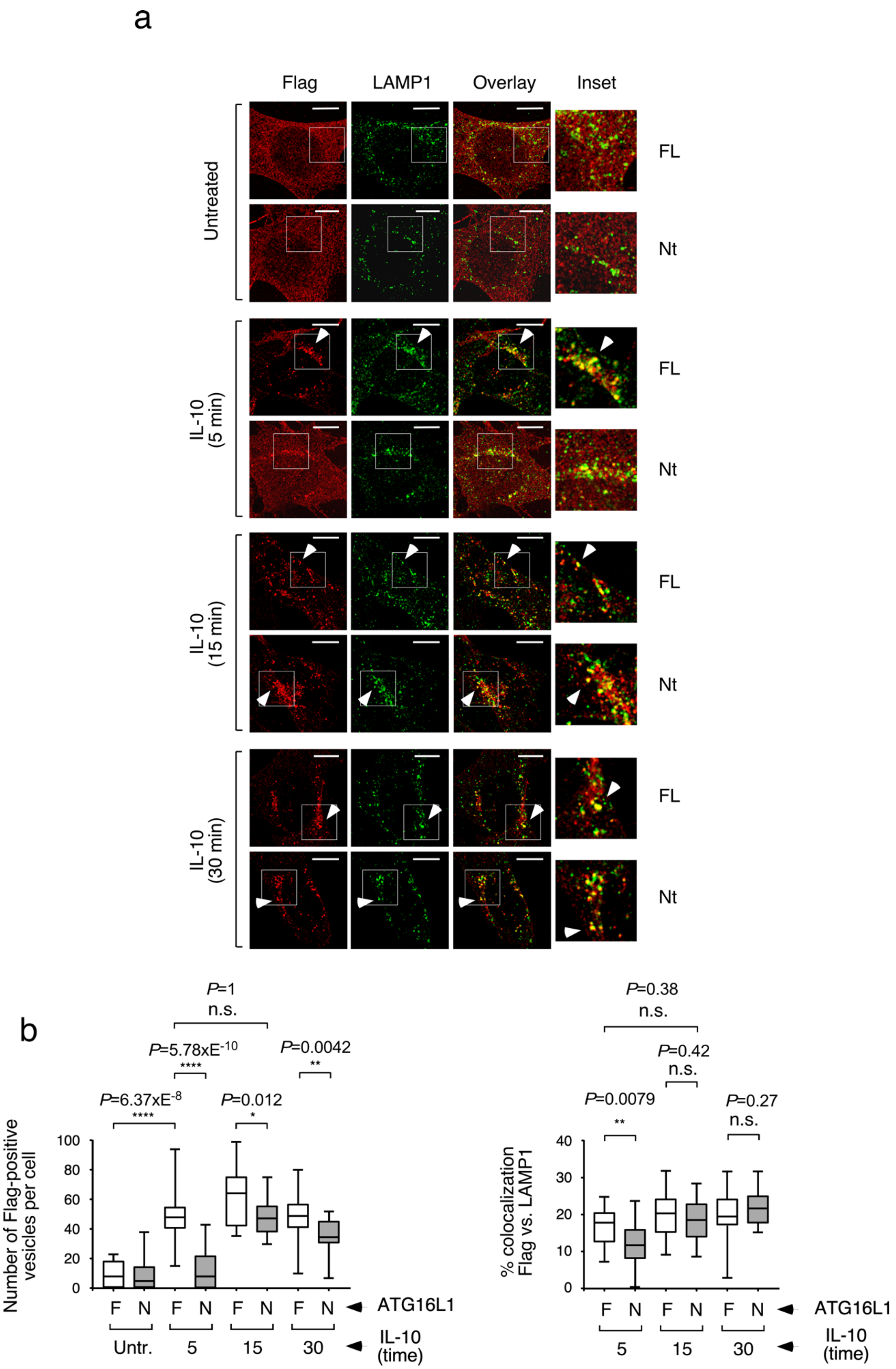
a
Flag
LAMP1
Overlay
Inset
Untreated
FL
Nt
IL-10 (5 min)
FL
Nt
IL-10 (15 min)
FL
Nt
IL-10 (30 min)
FL
Nt
b
P=1
n.s.
P=5.78xE-10
****
P=0.0042
**
P=6.37xE-8
****
P=0.012
*
Number of Flag-positive vesicles per cell
100
80
60
40
20
0
F N F N F N F N
ATG16L1
Untr. 5 15 30
IL-10 (time)
P=0.38
n.s.
P=0.42
n.s.
P=0.0079
**
P=0.27
n.s.
% colocalization Flag vs. LAMP1
40
30
20
10
0
F N F N F N
ATG16L1
5 15 30
IL-10 (time)

Supplementary Figure 11. Immunofluorescence assay to explore IL-10-induced IL-10RB endocytosis and intracellular trafficking to LAMP1-positive compartments. a, The indicated cells (FL or Nt) were continuously treated with IL-10 (50 ng/ml) for the shown times and fixed for immunofluorescence with anti-Flag (IL-10RB, red) and anti-LAMP1 (green) antibodies. Shown are representative confocal pictures. Arrows indicate examples of colocalization events. **b,** Box-plots displaying quantification of the phenotypes observed in **a**. The left panel shows the number of Flag-positive vesicles per cell at different times after IL-10 treatment observed in this experiment. The right panel represents the percentage of colocalizing red (Flag) and green (LAMP1) signals per cell. Data are presented as box-plots where the central line represents the median value, the box shows percentiles 25-75 and the whiskers include the most extreme values. For both panels, n=20 cells except for untreated cells (n=10); (n.s: $P>0,05$; (*): $P<0.05$; (**): $P<0.01$; (****): $P<0.0001$, two-sided Student's *t*-test). Source data are provided as a Source Data file.

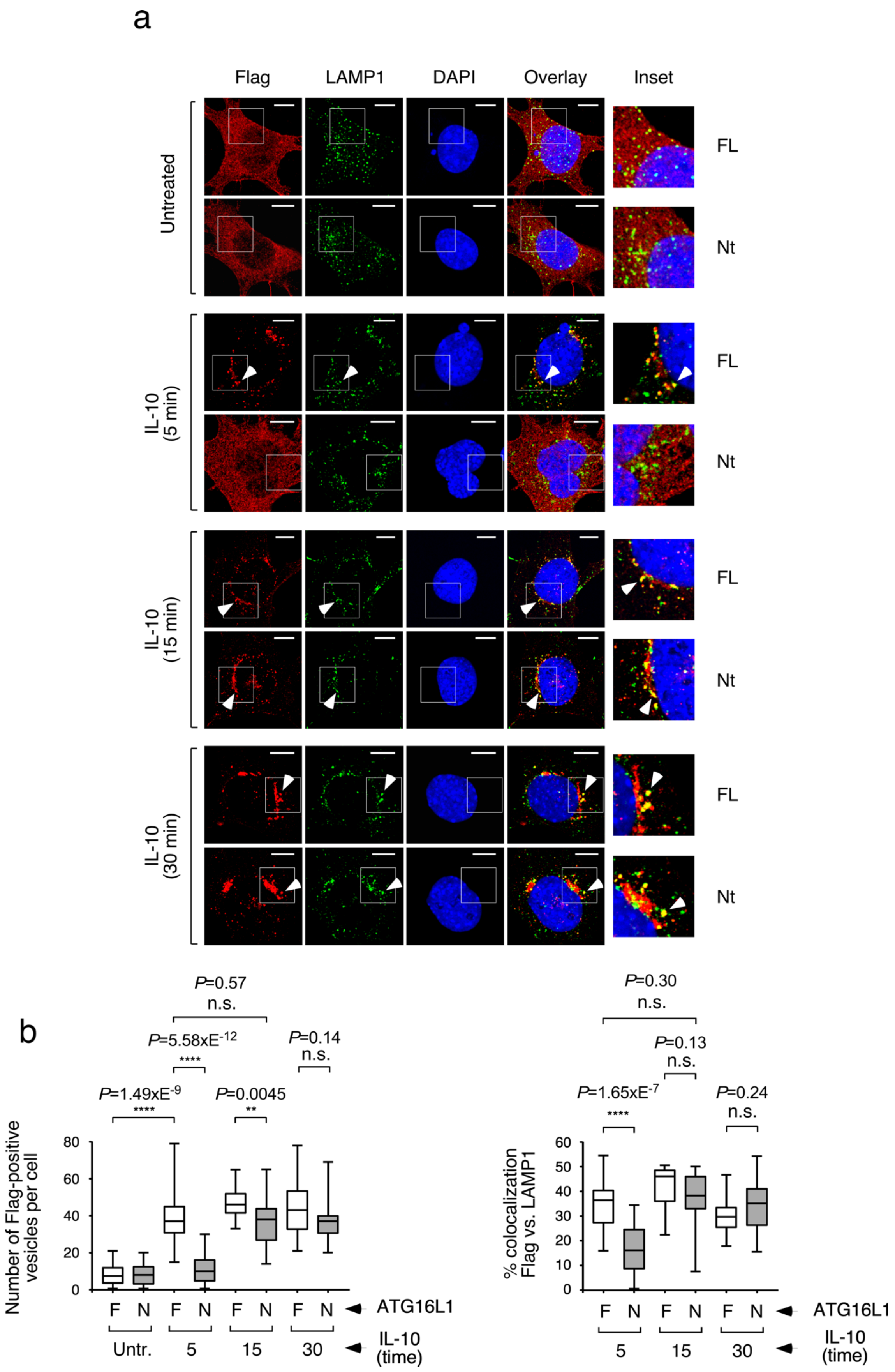
a
Flag
LAMP1
DAPI
Overlay
Inset
Untreated
IL-10 (5 min)
IL-10 (15 min)
IL-10 (30 min)
FL
Nt
b
P=0.57
n.s.
P=5.58xE-12
****
P=0.14
n.s.
P=1.49xE-9
****
P=0.0045
**
Number of Flag-positive vesicles per cell
F N F N F N F N
ATG16L1
Untr. 5 15 30
IL-10 (time)
P=0.30
n.s.
P=0.13
n.s.
P=1.65xE-7
****
P=0.24
n.s.
% colocalization Flag vs. LAMP1
F N F N F N
ATG16L1
5 15 30
IL-10 (time)

Supplementary Figure 12. Immunofluorescence pulse-chase assay to explore IL-10-induced IL-10RB endocytosis and intracellular trafficking to LAMP1-positive compartments. a, The indicated cells (FL or Nt) were pre-incubated with IL-10 (50 ng/ml; 30 min on ice), washed to remove IL-10 and then chased for the indicated times before fixation and staining with anti-Flag (IL-10RB, red) and anti-LAMP1 (green) antibodies. Shown are representative confocal pictures. Arrows indicate examples of colocalization events. **b,** Box-plots displaying quantification of the phenotypes observed in **a**. The left panel shows the number of Flag-positive vesicles per cell at different times after IL-10 treatment observed in this experiment. The right panel represents the percentage of colocalizing red (Flag) and green (LAMP1) signals per cell. Data are presented as box-plots where the central line represents the median value, the box shows percentiles 25-75 and the whiskers include the most extreme values. For both panels, n=25 cells except for untreated cells (n=14) (n.s: $P>0,05$; (**): $P<0.01$; (****): $P<0.0001$, two-sided Student's *t*-test). Source data are provided as a Source Data file.

a

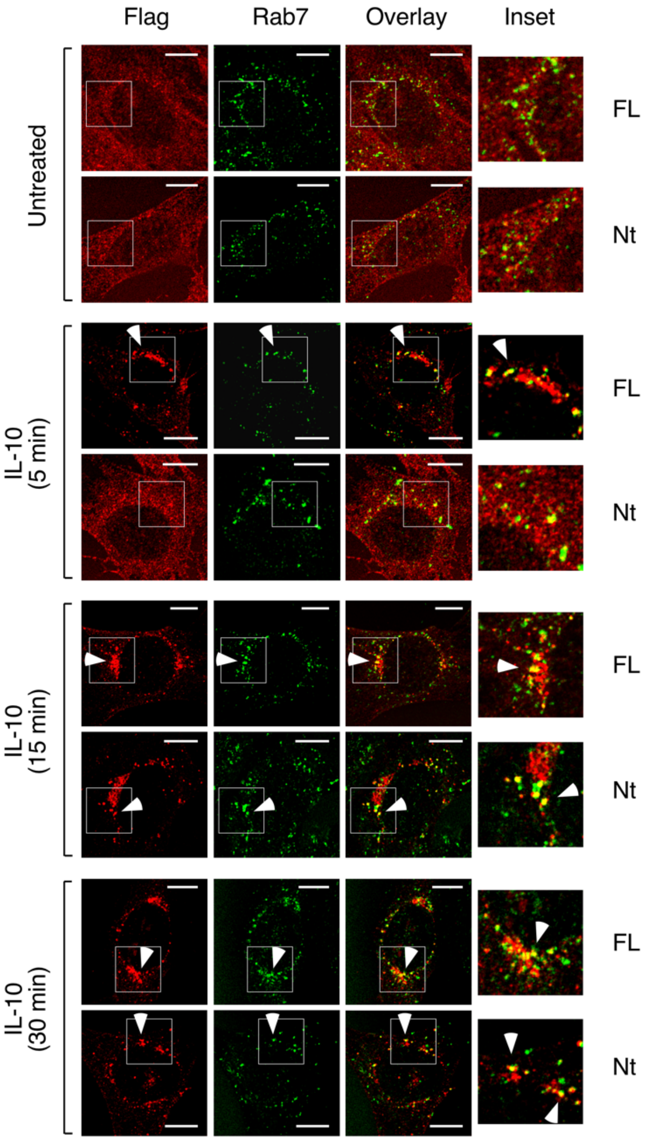
Flag
Rab7
Overlay
Inset
Untreated
IL-10 (5 min)
IL-10 (15 min)
IL-10 (30 min)
FL
Nt

b

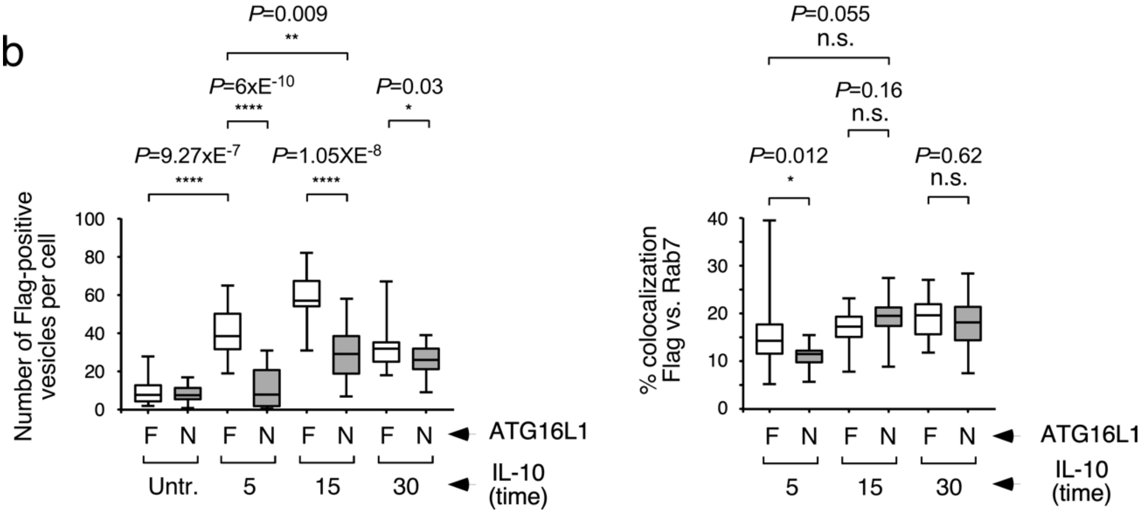
P=0.009
**
P=6xE-10
****
P=0.03
*
P=9.27xE-7
****
P=1.05XE-8
****
Number of Flag-positive vesicles per cell
F N F N F N F N
ATG16L1
Untr. 5 15 30
IL-10 (time)
P=0.055
n.s.
P=0.16
n.s.
P=0.012
*
P=0.62
n.s.
% colocalization Flag vs. Rab7
F N F N F N
ATG16L1
5 15 30
IL-10 (time)

Supplementary Figure 13. Immunofluorescence assay to explore IL-10-induced IL-10RB endocytosis and intracellular trafficking to Rab7-positive compartments. a, The indicated cells (FL or Nt) were continuously treated with IL-10 (50 ng/ml) for the shown times and fixed for immunofluorescence with anti-Flag (IL-10RB, red) and anti-Rab7 (green) antibodies. Shown are representative confocal pictures. Arrows indicate examples of colocalization events. **b,** Box-plots displaying quantification of the phenotypes observed in **a**. The left panel shows the number of Flag-positive vesicles per cell at different times after IL-10 treatment observed in this experiment. The right panel represents the percentage of colocalizing red (Flag) and green (Rab7) signals per cell. Data are presented as box-plots where the central line represents the median value, the box shows percentiles 25-75 and the whiskers include the most extreme values. For both panels, n=20 cells except for untreated cells (n=14); n.s: P>0,05; (*): P<0.05; (**): P<0.01; (****): P<0.0001, two-sided Student's *t*-test. Source data are provided as a Source Data file.

a

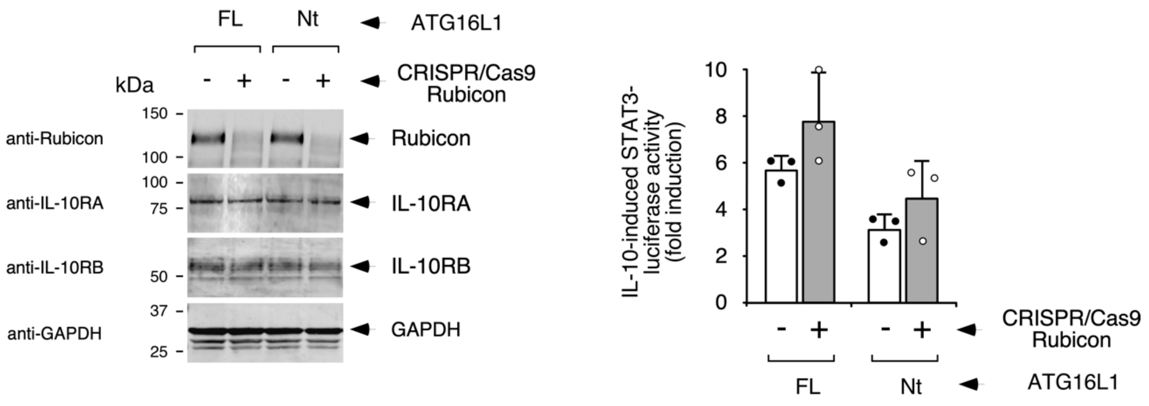
FL
Nt
ATG16L1
kDa
- + - +
CRISPR/Cas9 Rubicon
150
100
anti-Rubicon
Rubicon
100
75
anti-IL-10RA
IL-10RA
anti-IL-10RB
50
IL-10RB
37
25
anti-GAPDH
GAPDH
IL-10-induced STAT3-luciferase activity (fold induction)
10
8
6
4
2
0
- + - +
CRISPR/Cas9 Rubicon
FL
Nt
ATG16L1

b

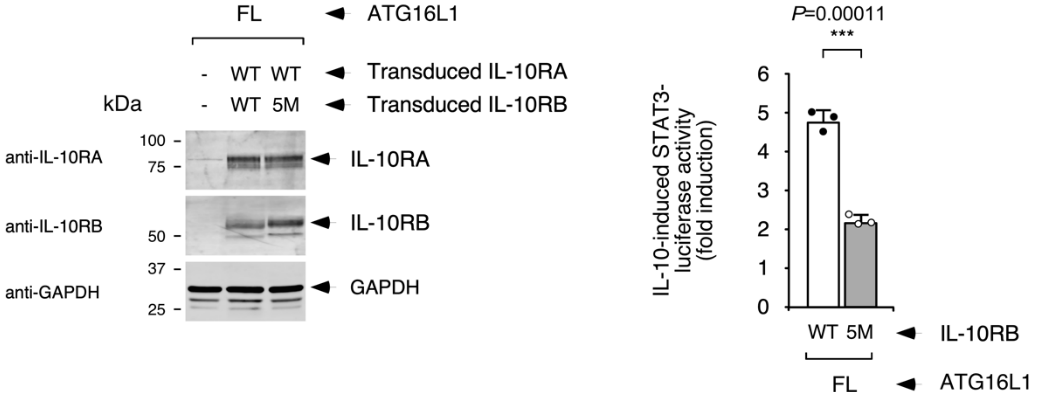
FL
ATG16L1
- WT WT
Transduced IL-10RA
kDa
- WT 5M
Transduced IL-10RB
100
75
anti-IL-10RA
IL-10RA
anti-IL-10RB
50
IL-10RB
37
25
anti-GAPDH
GAPDH
P=0.00011
***
IL-10-induced STAT3-luciferase activity (fold induction)
6
5
4
3
2
1
0
WT 5M
IL-10RB
FL
ATG16L1

Supplementary Figure 14. a, Analysis of IL-10 signalling in FL and Nt cells depleted of Rubicon. FL- and Nt-expressing MEFs (as indicated) were transduced with lentiviral control (-) or Rubicon-specific (+) CRISPR/Cas9 constructs, selected in blasticidin (2 ug/ml) for 6 days, lysed to evaluate the expression of the indicated molecules by Western-blotting (left panel) and tested for IL-10-induced STAT3-luciferase activity (50 ng/ml, 4 h; right panel). Data are expressed as means of fold induction of luciferase activity -/+ s.d. of 3 independent experiments each including triplicate experimental points (n=3). **b, Analysis of IL-10 signalling in cells expressing mutated IL-10RB (5M).** MEFs harbouring STAT3, the STAT3-luciferase reporter and FL ATG16L1 were transduced with retroviral constructs expressing IL-10RA and IL-10RB (WT or mutated (5M), as indicated), lysed 3 days later for Western-blotting against the indicated molecules (left panel) and tested for IL-10-induced STAT3-luciferase activity (50 ng/ml, 4 h; right panel). Data are expressed as means of fold induction of luciferase activity -/+ s.d. of triplicate experimental points (***): $P<0.001$, two-sided Student's *t*-test. Source data are provided as a Source Data file.

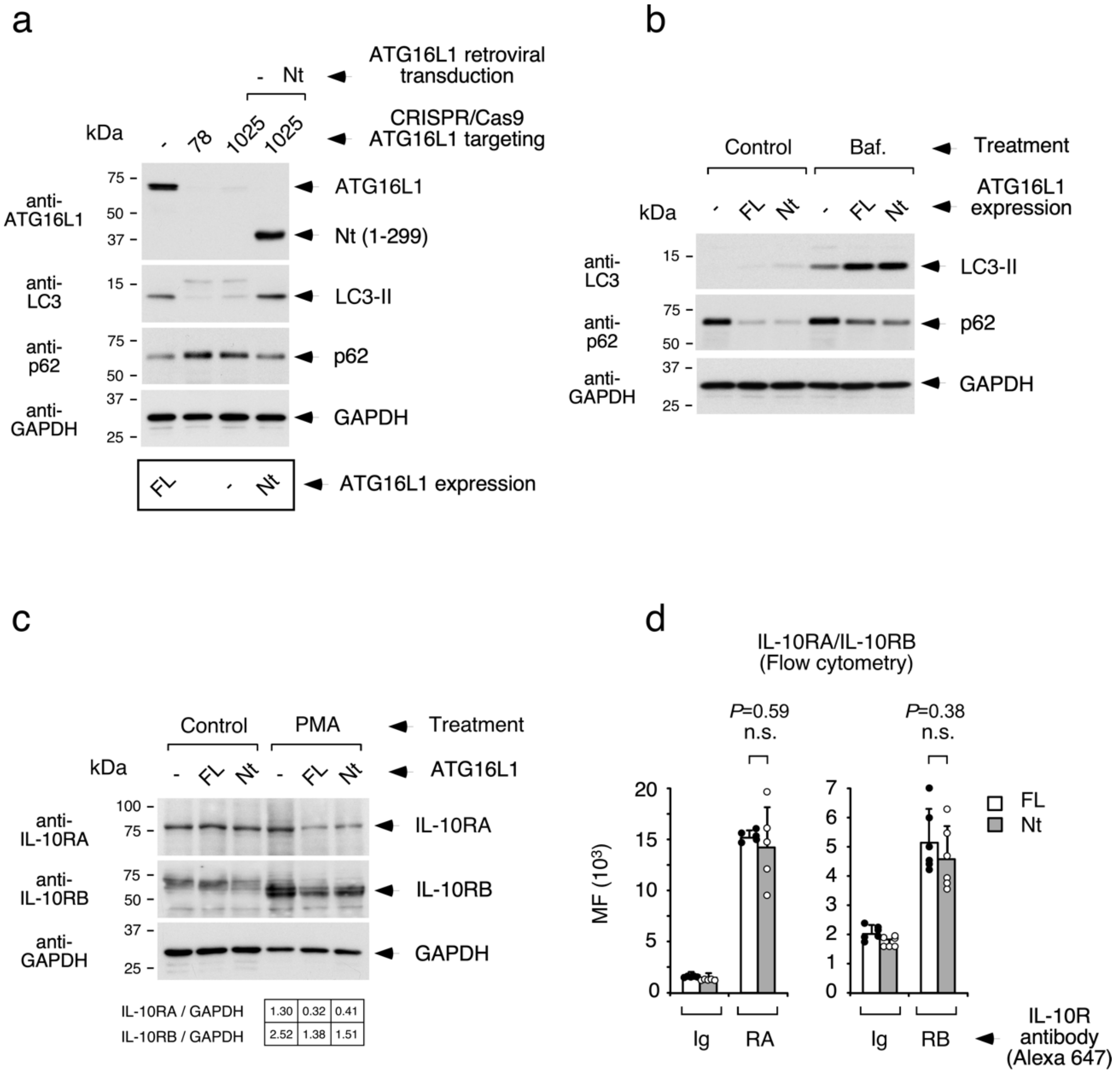
a
ATG16L1 retroviral transduction
- Nt
CRISPR/Cas9 ATG16L1 targeting
kDa
- 78 1025 1025
anti-ATG16L1
ATG16L1
Nt (1-299)
anti-LC3
LC3-II
anti-p62
p62
anti-GAPDH
GAPDH
FL - Nt
ATG16L1 expression
b
Control
Baf.
Treatment
ATG16L1 expression
kDa
- FL Nt - FL Nt
anti-LC3
LC3-II
anti-p62
p62
anti-GAPDH
GAPDH
c
Control
PMA
Treatment
kDa
- FL Nt - FL Nt
ATG16L1
anti-IL-10RA
IL-10RA
anti-IL-10RB
IL-10RB
anti-GAPDH
GAPDH
IL-10RA / GAPDH 1.30 0.32 0.41
IL-10RB / GAPDH 2.52 1.38 1.51
d
IL-10RA/IL-10RB (Flow cytometry)
P=0.59 n.s.
P=0.38 n.s.
MF (10³)
FL
Nt
Ig RA
Ig RB
IL-10R antibody (Alexa 647)

Supplementary Figure 15. Characterization of THP1 cells engineered to test the role of the WDD in IL-10 signalling through endogenous IL-10Rs. a, CRISPR/Cas9-induced depletion of endogenous ATG16L1 and restoration with a retroviral construct expressing the Nt domain (aminoacids 1-299). THP1 cells were transduced with lentiviral CRISPR/Cas9 constructs harbouring the indicated RNA guides directed to endogenous ATG16L1 (78: sgRNA recognizes positions 78-97 from the first coding nucleotide in ATG16L1 mRNA; 1025: sgRNA recognizes positions 1025-1044; (-): empty vector). Cells targeted with the 1025 guide were restored with a retroviral construct expressing the Nt domain of ATG16L1 (residues 1-299; positions 1-897 in ATG16L1 mRNA, not overlapping with the target sequence of the 1025 CRISPR/Cas9 guide). Cells were lysed for Western-blotting with the indicated antibodies. The nomenclature of cells used in subsequent assays is shown below the Western-blot panels (ATG16L1 expression). **b,** Evaluation of the autophagic flux in THP1 cells expressing endogenous ATG16L1 (FL) or ectopic Nt domain (Nt). The indicated cells were treated with bafilomycin A1 (75 nM; 6h) and lysed for Western-blotting with the indicated antibodies. **c,** Endogenous IL-10R expression in engineered THP1 cells. The indicated THP1 strains were incubated with PMA (125 ng/ml; 48 h) or left untreated (as shown), and lysed for Western-blotting with the indicated antibodies. Corrected densitometric quantifications are shown at the bottom of the Western-blot images. **d,** Surface expression levels of IL-10Rs. The indicated cells were incubated with PMA (125 ng/ml; 48 h) and subjected to flow cytometry with irrelevant (Ig) or specific anti-IL-10RA and -IL-10RB antibodies (as indicated). Shown are the average means of fluorescence (MF) from quintuplicate or sextuplicate experimental points -/+ s.d. (n=5 or n=6) for IL-10RA or IL-10RB respectively; n.s. *P*-value>0,05, two-sided Student's *t*-test). A scheme of the gating and quantification strategy is provided in Supplementary Figure 17. Source data are provided as a Source Data file.

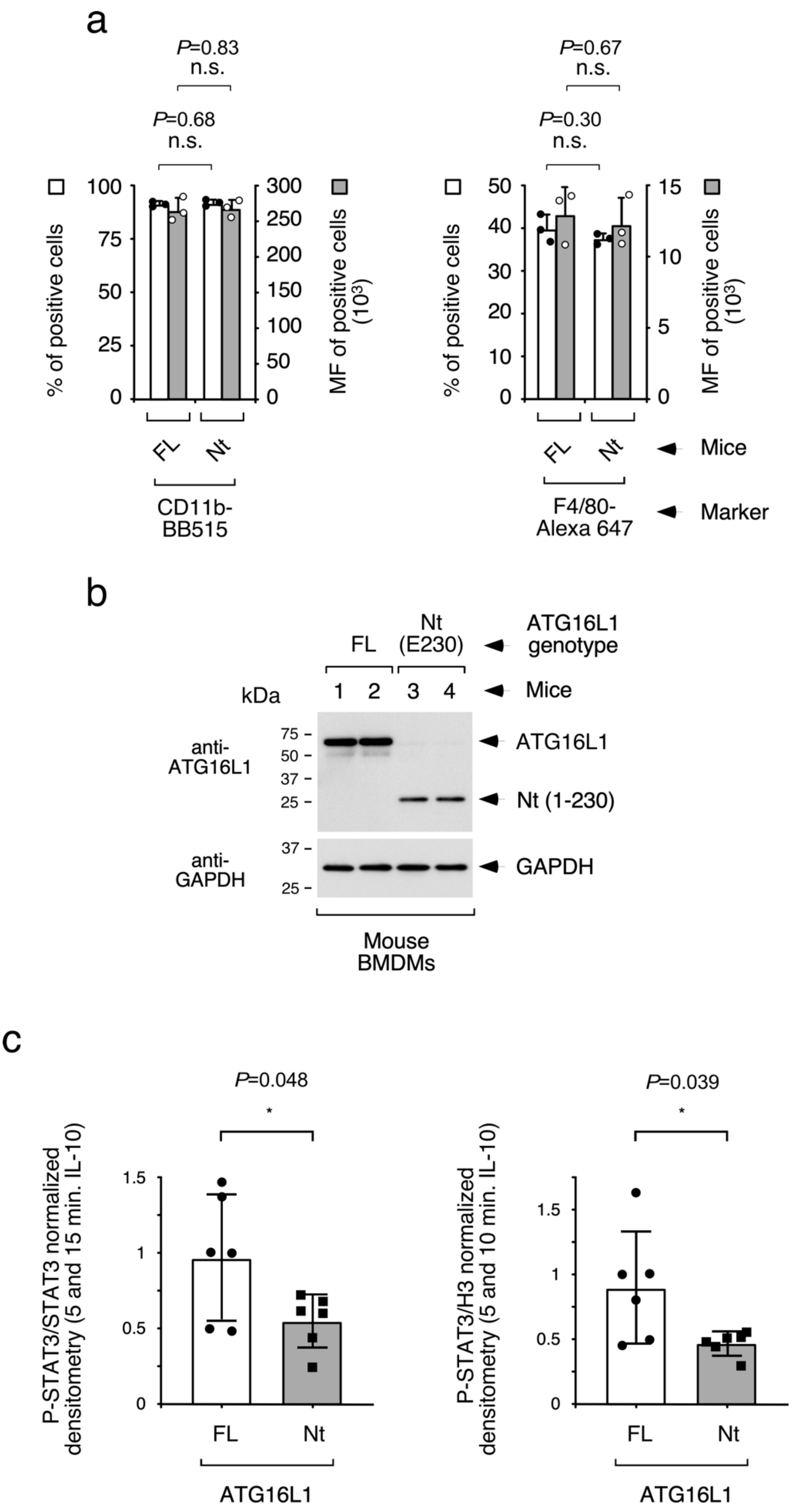
a
P=0.83
n.s.
P=0.68
n.s.
% of positive cells
MF of positive cells (10³)
100
75
50
25
0
300
250
200
150
100
50
0
FL
Nt
CD11b-BB515
P=0.67
n.s.
P=0.30
n.s.
50
40
30
20
10
0
15
10
5
0
F4/80-Alexa 647
Mice
Marker
b
FL
Nt (E230)
ATG16L1 genotype
kDa
1 2 3 4
Mice
anti-ATG16L1
75
50
37
25
ATG16L1
Nt (1-230)
anti-GAPDH
37
25
GAPDH
Mouse BMDMs
c
P=0.048
*
P-STAT3/STAT3 normalized densitometry (5 and 15 min. IL-10)
1.5
1
0.5
0
ATG16L1
P=0.039
*
P-STAT3/H3 normalized densitometry (5 and 10 min. IL-10)

Supplementary Figure 16. Characterization of BMDMs isolated from wild-type and E230 mice. a, Expression of the differentiation markers CD11 and F4/80. Bone marrow cells isolated from the indicated mice (FL: wild-type; Nt: E230) were differentiated in vitro (M-CSF 20 ng/ml; 6 days) and subjected to flow cytometry with specific anti-CD11b (left panel) and anti-F4/80 (right panel) antibodies. Shown are the average percentage of positive cells with respect to a control antibody (left axis, white bars, solid dots) and the average mean of fluorescence of the positive cells (MF, right axis, grey bars, white dots) from triplicate experimental points -/+ s.d. (n=3; n.s.: *P*-value>0,05, two-sided Student's *t*-test). A scheme of the gating and quantification strategy is provided in Supplementary Figure 17. **b,** Expression levels of ATG16L1 in wild-type and E230 BMDMs. BMDMs were obtained as in **a** from 2 wild-type (FL) and 2 E230 (Nt) mice (as indicated). Cells were lysed for Western-blotting against the shown molecules. **c,** Statistical analysis of nuclear P-STAT3 densitometric signals induced by IL-10, as shown in Fig. 6c. Crude densitometric values were internally normalized as the percentage of the highest value per series. Numbers for P-STAT3 were then normalized against those obtained for total STAT3 (left graph) or H3 (right graph), and the data corresponding to the 5 and 15 min time points were pooled together and evaluated for statistical significance. Shown are mean values -/+ s.d. (n=6; (*): *P*-value<0,05, two-sided Student's *t*-test). Source data are provided as a Source Data file.

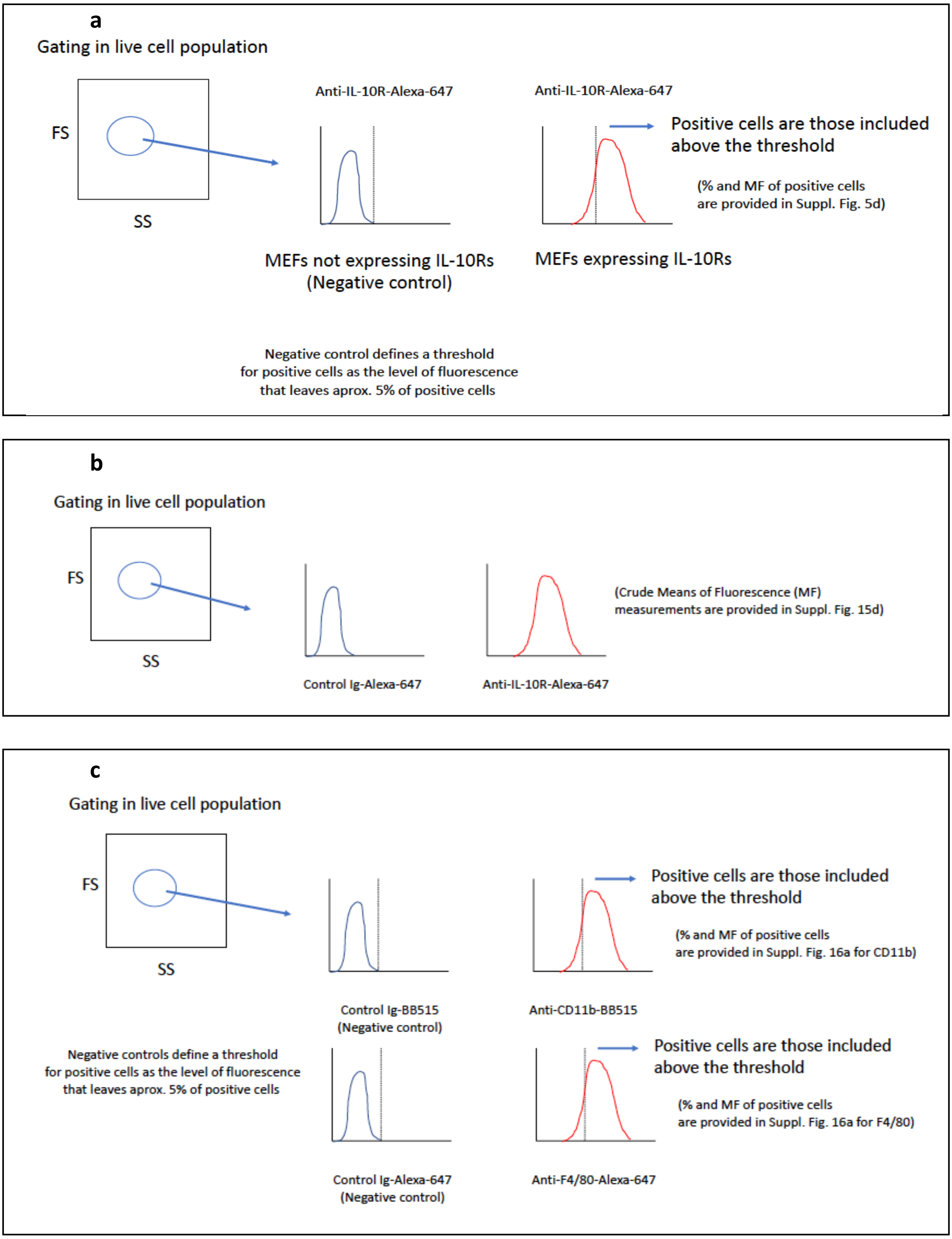
a
Gating in live cell population
FS
SS
Anti-IL-10R-Alexa-647
Anti-IL-10R-Alexa-647
Positive cells are those included above the threshold
(% and MF of positive cells are provided in Suppl. Fig. 5d)
MEFs not expressing IL-10Rs (Negative control)
MEFs expressing IL-10Rs
Negative control defines a threshold for positive cells as the level of fluorescence that leaves aprox. 5% of positive cells
b
Gating in live cell population
FS
SS
(Crude Means of Fluorescence (MF) measurements are provided in Suppl. Fig. 15d)
Control Ig-Alexa-647
Anti-IL-10R-Alexa-647
c
Gating in live cell population
FS
SS
Positive cells are those included above the threshold
(% and MF of positive cells are provided in Suppl. Fig. 16a for CD11b)
Control Ig-BB515 (Negative control)
Anti-CD11b-BB515
Negative controls define a threshold for positive cells as the level of fluorescence that leaves aprox. 5% of positive cells
Positive cells are those included above the threshold
(% and MF of positive cells are provided in Suppl. Fig. 16a for F4/80)
Control Ig-Alexa-647 (Negative control)
Anti-F4/80-Alexa-647

Supplementary Figure 17. Gating schemes of flow cytometry analyses shown in Supplementary Figures 5d, 15d and 16a. Cells were in all cases gated at the FS/SS plot to select live cells and remove debris. **a, Scheme for Supplementary Figure 5d.** MEFs not expressing or expressing IL-10Rs were stained with IL-10R-specific antibodies. Cells lacking IL-10Rs were used as a negative control to define a threshold leaving approximately 5% of positive cells. Supplementary Figure 5d shows the % and mean of fluorescence (MF) of the cells expressing IL-10Rs that show fluorescence intensity levels above the established threshold. **b, Scheme for Supplementary Figure 15d.** THP1 cells were stained with a control Ig-Alexa-647 or specific anti-IL-10Rs-Alexa-647 antibodies. Supplementary Figure 15d shows crude values of fluorescence intensity (MF) obtained in both conditions. **c, Scheme for Supplementary Figure 16a.** Differentiated BMDMs were stained with irrelevant Ig-BB515 or Ig-Alexa-647 control antibodies, and with specific anti-CD11b-BB515 or anti-F4/80-Alexa-647 antibodies, respectively. The irrelevant controls were used to define thresholds leaving approximately 5% of positive cells. Supplementary Figure 16a shows the % and mean of fluorescence (MF) of the cells stained with anti-CD11b or anti-F4/80 that show fluorescence intensity levels above the established threshold.

SUPPLEMENTARY TABLES 1 TO 5

Supplementary Table 1. Oligonucleotides for PCR

IL-2Rg-Hind3-Kozak-fw	5’ gggcccaagcttgccaccatgttgaagccatcattaccattcacatcc 3’
IL-2Rg-Bsph1-rev	5’ cccgggtcatgacggtttcaggctttagggtgtaacatgg 3’
IL-10RB-Hind3-Kozak-fw	5’ gggcccaagcttgccaccatggcgtggagccttgggagctggctggg 3’
IL-10RB-Bsph1-rev	5’ cccgggtcatgacgctttggggcccctgcccaggcggggtc 3’
IL-4RA-EcoR1-Kozak-fw	5’ gggcccgaattcgccaccatggggtggctttgctctgggctcctgttc 3’
IL-4RA-Bsph1-rev	5’ cccgggtcatgacagagaccctcatgtatgtgggtcccac 3’
IL-6RB-Hind3-Kozak-fw	5’ gggcccaagcttgccaccatgttgacgttgcagacttggctagtgc 3’
IL-6RB-Bsph1-rev	5’ cccgggtcatgacctgaggcatgtagccgccttgccgtac 3’
IL-12RB2-Hind3-Kozak-fw	5’ gggcccaagcttgccaccatggcacatacttttagaggatgctcattg 3’
IL-12RB2-Bsph1-rev	5’ cccgggtcatgacgagcatgagggagtcacacctcatctttaac 3’
IL-20RA-Hind3-Kozak-fw	5’ gggcccaagcttgccaccatgcgggctcccggccgcccggccctgc 3’
IL-20RA-Bsph1-rev	5’ cccgggtcatgacgttttccatctgcacatataaccccc 3’
IL-21R-EcoR1-Kozak-fw	5’ gggcccgaattcgccaccatgccgcgtggctgggccgccccccttgc 3’
IL-21R-Bsph1-rev	5’ cccgggtcatgacgctggcctggggtccagggctcgaaag 3’
IL18R-EcoR1-Kozak-fw	5’ gggcccgaattcgccaccatgaattgtagagaattacccttgaccc 3’
IL18R-Bsph1-Stop-Not1-rev	5’ cccggggcggccgctttagctcatgacagactcggaaagaacaggcaagacttccgg 3’
IL18RAP-Hind3-Kozak-fw	5’ gggcccaagcttgccaccatgctctgtttgggctggatatttctttggc 3
IL18RAP-Bsph1-Stop-Not1-rev	5’ cccggggcggccgctttagctcatgacccattccttaggctgggagctcctccc 3
IL-31RA-Hind3-Kozak-fw	5’ gggcccaagcttgccaccatgaagctctctccccagccttcatgtgttaacctg 3’
IL-31RA-Bsph1-Stop-Not1-rev	5’ cccggggcggccgctttagctcatgacgacttctcccttggtgtgctctggaag 3’
IL-33R-Hind3-Kozak-fw	5’ gggcccaagcttgccaccatggggttttggatcttagcaattctcac 3’
IL-33R-Bsph1-rev	5’ cccgggtcatgacttgcttctgggcagccaagggagtcaaac 3’
IL-17RD-Pci1-fw	5’ gggcccacatgtcctggcagggagtggggccagccagcag 3’
IL-17RD-Bsph1-Stop-Not1-rev	5’ cccggggcggccgctttagctcatgaccaaaggggcgaccgcgtggagttcatcag 3’

Sequences of the oligonucleotides used to generate DNA constructs expressing tagged IL-Rs by PCR

Supplementary Table 2. Oligonucleotides for site-directed mutagenesis

IL-10RB-F292,L295A-TOP	5’ gtcggatgagaatgatgttgctgacaaggcaagtgtcattgcagaagac 3’
IL-10RB-F292,L295A-BOTTOM	5’ gtcttctgcaatgacacttgccttgtcagcaacatcattctcatccgac 3’
IL-10RB-F280,F281,F283A-(5M)-TOP	5’ ctcatcataacacacttctggctgcctccgctccattgtcggatgagaatgatg 3’
IL-10RB-F280,F281,F283A-(5M)-BOTTOM	5’ catcattctcatccgacaatggagcggaggcagccagaagtgtgttatgatgag 3’
IL-2Rg-F307,W310A-TOP	5’ gttactgaataccacgggaacgcttcggccgccagtggtgtgtctaagggac 3’
IL-2Rg-F307,W310A-BOTTOM	5’ gtcccttagacacaccactggcggccgaagcgttcccgtggtattcagtaac 3’
IL-2Rg-L296,L299,Y303A-(5M)-TOP	5’ cgaattcccaccctgaagaacgcagaggatgctgttactgaagcccacgggaacgcttcggccgcc 3’
IL-2Rg-L296,L299,Y303A-(5M)-BOTTOM	5’ ggcggccgaagcgttcccgtgggcttcagtaacagcatcctctgcgttcttcagggtgggaattcg 3’
IL-2Rg-L293,L296,L299,Y303A-(6M)-TOP	5’ gacgatgccccgaattcccaccgcgaagaacgcagaggatgctgttactgaagcccacgggaacgcttcggccgcc 3’
IL-2Rg-L293,L296,L299,Y303A-(6M)-BOTTOM	5’ ggcggccgaagcgttcccgtgggcttcagtaacagcatcctctgcgttcttcgcggtgggaattcggggcatcgtc 3’
IL-18R1 intron deletion-TOP	5’ caacagcacatcattgtataagaactgtaaaaagctactactgg 3’
IL-18R1 intron deletion-BOTTOM	5’ ccagtagtagctttttacagttcttatacaatgatgtgctgttg 3’

Sequences of the oligonucleotides used to generate new DNA constructs by site-directed mutagenesis

Supplementary Table 3. Oligonucleotides including the STAT3 operon

STAT3op-Hpa1-Xho1-TOP	5’ aacctcgagtgcttcccgaacgttgcttcccgaacgttgcttcccgaacgttgcttcccgaacgt 3’
STAT3op-Hpa1-Xho1-BOTT	5’ tcgaacgttcgggaagcaacgttcgggaagcaacgttcgggaagcaacgttcgggaagcactcgaggtt 3’

Sequences of the oligonucleotides used to multimerize the STAT3 enhancer to generate a retroviral STAT3-luciferase reporter system

Supplementary Table 4. Oligonucleotides for CRISPR/Cas9 guides

human ATG16 (78-97)-TOP	5’ caccggctgcagagacaggcgttcg 3’
human ATG16 (78-97)-BOTTOM	5’ aaaccgaacgcctgtctctgcagcc 3’
human ATG16L1 (1025-1044)-TOP	5’ caccgtggaccgcagggttaagctt 3’
human ATG16L1 (1025-1044)-BOTTOM	5’ aaacaagcttaaccctgcggtccac 3’
Mouse Rubicon (29-48)-TOP	5’ caccgagtttgtgaaagacattcgg 3’
Mouse Rubicon (29-48)-BOTTOM	5’ aaacccgaatgtctttcacaaactc 3’

Sequences of the oligonucleotides used to generate CRISPR/Cas9 sgRNAs to reduce the expression of ATG16L1 and Rubicon

Supplementary Table 5. Oligonucleotides for quantitative PCR

Human TNFa-fw	5' cctctctctaatcagccctctg 3'
Human TNFa-rev	5' gaggacctgggagtagatgag 3'
Human IL-6-fw	5' actcacctcttcagaacgaattg 3'
Human IL-6-rev	5' ccatctttggaaggttcaggttg 3'
Human bActin-fw	5' catgtacgttgctatccaggc 3'
Human bActin-rev	5' ctccttaatgtcacgcacgat 3'
Human GAPDH-fw	5' ggagcgagatccctccaaaat 3'
Human GAPDH-rev	5' ggctgttgtcatacttctcatgg 3'
Mouse Bcl3-fw	5' ccggaggccctttactacca 3'
Mouse Bcl3-rev	5' ggagtaggggtgagtaggcag 3'
Mouse TNFa-fw	5' ccctcacactcagatcatcttct 3'
Mouse TNFa-rev	5' gctacgacgtgggctacag 3'
Mouse bActin-fw	5' ggctgtattcccctccatcg 3'
Mouse bActin-rev	5' ccagttggtaacaatgccatgt 3'
Mouse GAPDH-fw	5' aggtcggtgtgaacggatttg 3'
Mouse GAPDH-rev	5' tgtagaccatgtagttgaggtca 3'

Sequences of the oligonucleotides used to measure mRNA levels by qPCR
