# Supplementary material for: Regulation of cytokine signaling through direct interaction between cytokine receptors and the ATG16L1 WD40 domain
Source: Nat Commun. 2020 Nov 20;11:5919. doi: 10.1038/s41467-020-19670-4 (PMC7679444; doi:10.1038/s41467-020-19670-4)
Supplement: Supplementary file 3 — Descriptions of Additional Supplementary Files [file 41467_2020_19670_MOESM3_ESM.pdf]

Descriptions of Additional Supplementary Files

Supplementary Data 1

Description: Complete list of Type-I transmembrane proteins including the WDD-binding motif in their intracellular domains. Motif identities for all molecules are shown in Supplementary Data 2.

Supplementary Data 2

Description: Motifs identified in the intracellular domains of Type-I transmembrane proteins shown in Supplementary Data 1.

Supplementary Data 3

Description: Complete list of cytokine receptors including the WDDbinding motif in their intracellular domains. Motif identities for each molecules are shown in Supplementary Data 4.

Supplementary Data 4

Description: Motifs identified in the intracellular domains of cytokine receptors shown in Supplementary Data 3.

Supplementary Data 5

Description: List of all human Type-I transmembrane proteins downloaded from the Uniprot server.
