# Supplementary material for: Regulation of cytokine signaling through direct interaction between cytokine receptors and the ATG16L1 WD40 domain
Source: Nat Commun. 2020 Nov 20;11:5919. doi: 10.1038/s41467-020-19670-4 (PMC7679444; doi:10.1038/s41467-020-19670-4)
Supplement: Supplementary file 9 — Reporting Summary [file 41467_2020_19670_MOESM9_ESM.pdf]

nature research

Corresponding author(s): Felipe X. Pimentel-Muiños

Last updated by author(s): Oct 5, 2020

Reporting Summary

Nature Research wishes to improve the reproducibility of the work that we publish. This form provides structure for consistency and transparency in reporting. For further information on Nature Research policies, see our Editorial Policies and the Editorial Policy Checklist.

Statistics

For all statistical analyses, confirm that the following items are present in the figure legend, table legend, main text, or Methods section.

n/a	Confirmed	
☐	☒	The exact sample size (*n*) for each experimental group/condition, given as a discrete number and unit of measurement
☐	☒	A statement on whether measurements were taken from distinct samples or whether the same sample was measured repeatedly
☐	☒	The statistical test(s) used AND whether they are one- or two-sided<br>*Only common tests should be described solely by name; describe more complex techniques in the Methods section.*
☒	☐	A description of all covariates tested
☒	☐	A description of any assumptions or corrections, such as tests of normality and adjustment for multiple comparisons
☐	☒	A full description of the statistical parameters including central tendency (e.g. means) or other basic estimates (e.g. regression coefficient) AND variation (e.g. standard deviation) or associated estimates of uncertainty (e.g. confidence intervals)
☐	☒	For null hypothesis testing, the test statistic (e.g. *F*, *t*, *r*) with confidence intervals, effect sizes, degrees of freedom and *P* value noted<br>*Give P values as exact values whenever suitable.*
☒	☐	For Bayesian analysis, information on the choice of priors and Markov chain Monte Carlo settings
☒	☐	For hierarchical and complex designs, identification of the appropriate level for tests and full reporting of outcomes
☒	☐	Estimates of effect sizes (e.g. Cohen's *d*, Pearson's *r*), indicating how they were calculated

Our web collection on statistics for biologists contains articles on many of the points above.

Software and code

Policy information about availability of computer code

Data collection	Accuri C6 software (BD Biosciences; version 1.0.264.21), LAS AF X Core Suite (version 2.7.3.9723)-confocal colocalization statistics (Leica Microsystems), qPCR (BioRad; iQ5TM 2.0 Standard Edition Optical System Software), BreakingCas server (CNB, Madrid, Spain) sgRNA design (no version available), GenePix Pro 6.0 software for GenePix 4000B microarray scanner, PrimerBank Primer Search on-line tool (no version available)
Data analysis	Microsoft Excel 1902, GraphPad Prism 7.04, ScanProsite tool (Expasy, Bioinformatics Resource Portal; no version available)

For manuscripts utilizing custom algorithms or software that are central to the research but not yet described in published literature, software must be made available to editors and reviewers. We strongly encourage code deposition in a community repository (e.g. GitHub). See the Nature Research guidelines for submitting code & software for further information.

Data

Policy information about availability of data

All manuscripts must include a data availability statement. This statement should provide the following information, where applicable:

- Accession codes, unique identifiers, or web links for publicly available datasets
- A list of figures that have associated raw data
- A description of any restrictions on data availability

Source data and uncropped versions of the most relevant Western-blot images for all main and supplementary figures (except Supplementary Figure 17) are provided as a Source Data file. Details on the gating and quantification strategies are provided in Supplementary Figure 17. The database of human Type-I transmembrane proteins used to search for the WDD-binding motif is available as Supplementary Data 5 in a Fasta format. Information about how to retrieve this database from the public Uniprot sequence bank is provided in the Methods section (under Prosite Comparisons). All relevant data are available from the authors upon reasonable request.

Field-specific reporting

Please select the one below that is the best fit for your research. If you are not sure, read the appropriate sections before making your selection.

- [x] Life sciences
- [ ] Behavioural & social sciences
- [ ] Ecological, evolutionary & environmental sciences

For a reference copy of the document with all sections, see nature.com/documents/nr-reporting-summary-flat.pdf

Life sciences study design

All studies must disclose on these points even when the disclosure is negative.

Sample size	Sample sizes were chosen based on the general practice in the field indicating that at lease triplicate experimental replicas (for STAT3-luciferase IL-10 signalling studies; Gemelli et al. BBA, 1843:955. 2014), n=20 cells (intracellular trafficking analyses; Boada-Romero et al. Nat. Commun. 7:11821. 2016) and n=3 mice (studies of IL-10 anti-inflammatory activity in macrophages; Kuwata et al. Blood, 102:4123. 2003; Nakamura et al. Nat. Commun. 6:7847. 2015) suffice to reveal significant differences in similar studies. Sample sizes that we chose throughout our manuscript were considered sufficient as long as the differences/no differences detected were statistically solid (significant) and reproducible
Data exclusions	Some Western-blots were excluded owing to poor quality. Some of the datasets involving isolation and experimentation with BMDMs were excluded because the final population did not show induction of pro-inflammatory cytokines by lps and/or no inhibition provided by IL-10 in wild-type cells. This occurred mainly during the initial optimization of the technique, and we think it was due to our initial lack of expertise in purification and handling (in vitro differentiation) of BMDM samples
Replication	All results were replicated at least three times with similar results
Randomization	Mice were chosen randomly as long as the experimental groups (WT and E320) were comparable in terms of sex and age
Blinding	Numbers of intracellular vesicles were blindly scored by counting the relevant events on confocal pictures. For all other experiments, group allocation and data collection were also performed blindly

Reporting for specific materials, systems and methods

We require information from authors about some types of materials, experimental systems and methods used in many studies. Here, indicate whether each material, system or method listed is relevant to your study. If you are not sure if a list item applies to your research, read the appropriate section before selecting a response.

Materials & experimental systems

n/a	Involved in the study	
[ ]	[x]	Antibodies
[ ]	[x]	Eukaryotic cell lines
[x]	[ ]	Palaeontology and archaeology
[ ]	[x]	Animals and other organisms
[x]	[ ]	Human research participants
[x]	[ ]	Clinical data
[x]	[ ]	Dual use research of concern

Methods

n/a	Involved in the study	
[x]	[ ]	ChIP-seq
[ ]	[x]	Flow cytometry
[x]	[ ]	MRI-based neuroimaging

Antibodies

Antibodies used	Antibodies, their suppliers, references and dilutions are provided in the Methods section:<br>Western blotting:<br>anti-GST (1/1000, rabbit polyclonal, Cell Signaling 2622), anti-HA (1/1000, 16B12, mouse mAb, Babco Biolegend 901501), anti-AU1 (1/1000, rabbit polyclonal, Thermo PA1-26548), anti-Flag (1/1000, M2, mouse mAb, Sigma F3165), anti-GFP (1/5000, B34, mouse mAb, BioLegend 902601), anti-ATG16L1 (1/2000, 1F12, mouse mAb, MBL M150-3), anti-IL-10RA (1/500, A3, mouse mAb, Santa Cruz sc-365374), anti-IL-10RB (1/500, F6, mouse mAb, Santa Cruz sc-271969), anti-phospho-STAT3-Y705 (1/1000, rabbit polyclonal, Cell Signaling 9131), anti-STAT3 (1/1000, mouse mAb, Cell Signaling 9139), anti-IL-10 (1/1000, D13A11, rabbit mAb, Cell Signaling 12163), anti-Histone-3 (1/1000, D1H2, rabbit mAb, Cell Signaling 4499), anti-LC3 (1/1000, mouse mAb, MBL M186-3), anti-p62 (THPs, 1/1000, mouse mAb, BD 610833; MEFs 1/1000, rabbit polyclonal, MBL PM045), anti-Rubicon (1/1000, D9F7, rabbit mAb, Cell Signaling 8465), anti-GAPDH (1/10000, 6C5, mouse mAb, Abcam ab8245)<br>Immunoprecipitation:<br>Anti-Flag (1/150, D6W5B, rabbit mAb, Cell Signaling 2368), anti-IL-10RB (1/50, F6, mouse mAb, Santa Cruz sc-271969), anti-ATG16L1 (1/100, rabbit polyclonal, MBL PM-040)<br>Immunofluorescence:

	anti-Flag (in most cases rabbit polyclonal (1/2000, Cell Signaling 2368); mouse monoclonal (1/100, Sigma F3165) for co-staining with anti-EEA1), anti-EEA1 (1/750, EPR4245, rabbit mAb, Abcam ab109110), anti-LC3 (1/200, 5F10, mouse mAb, NanoTools 0231-100), anti-LAMP1 (1/200, EPR21026, rabbit mAb, Abcam, ab208943), anti-Rab7 (1/100, EPR7589, rabbit mAb, Abcam, ab137029).
Validation	Suppliers and antibodies were chosen based on how much validation information was available in their website. Antibodies were not systematically validated in-house, but all of them provided predictable results in terms of molecular weight of the recognized bands, patterns of intracellular staining and so on. Anti-IL-10R antibodies did not recognize any band in cells lacking IL-10Rs. Anti-ATG16L1 antibodies did not recognize any relevant band in ATG16L1-/- cells. Anti-IL10 and P-STAT3 antibodies did not recognize any bands in untreated cells.

Eukaryotic cell lines

Policy information about cell lines

Cell line source(s)	ATCC (HEK-293T, HeLa, THP-1). The ATG16L1-/- HCT116 cell line was obtained from Dr. David Boone (Indiana University School of Medicine, South Bend, Indiana, USA), and Atg16L1-/- MEFs were kindly shared by Dr. Ramnik Xavier (Broad Institute MIT and Harvard, USA).
Authentication	Cell lines were not authenticated in house. We trusted the supplier (ATCC). We validated ATG16L1 deficiency of ATG16L1-/- HCT116 and MEFs by anti-ATG16L1 Western-blotting
Mycoplasma contamination	Cells were tested for mycoplasma contaminationand none of them was found to be contaminated
Commonly misidentified lines (See ICLAC register)	No commonly misidentified cell lines were used in this study

Animals and other organisms

Policy information about studies involving animals; ARRIVE guidelines recommended for reporting animal research

Laboratory animals	Mus musculus (laboratory mice), C54BL/6, males and females between 4-8 weeks old. Mice were maintained in ventilated racks in a Specific Pathogen-Free facility under controlled temperature (23° C), humidity (50%) and light/dark cycle (12 h/12 h) conditions, strictly following European Union regulations
Wild animals	No wild animals were used in this study
Field-collected samples	No field collected samples were used in this study
Ethics oversight	All procedures were evaluated and approved by the Ethics and Animal Wellbeing Committees of the University of Salamanca and the Junta de Castilla y Leon local government.

Note that full information on the approval of the study protocol must also be provided in the manuscript.

Flow Cytometry

Plots

Confirm that:

- [x] The axis labels state the marker and fluorochrome used (e.g. CD4-FITC).
- [x] The axis scales are clearly visible. Include numbers along axes only for bottom left plot of group (a 'group' is an analysis of identical markers).
- [x] All plots are contour plots with outliers or pseudocolor plots.
- [x] A numerical value for number of cells or percentage (with statistics) is provided.

Methodology

Sample preparation	MEFs, THP1 and BMDMs were detached using the trypsin-free Accutase Solution (Sigma), resuspended in culture medium containing 0,1% azide and a 1:100 dilution of mouse or rat immunoglobulins (Sigma) as blocking agents, and incubated for 30 min on ice. The specific antibodies were then added to the cell suspension at a 1:100 dilution, and an additional incubation was carried out on ice for 30 min. Cells were then washed and analysed in an Accuri C6 (BD BioSciences) flow cytometer. For isolation of BMDMs, mice were sacrificed using a CO2 chamber and immediately dissected to isolate bone marrow cells by flushing the tibias and femurs with a 23G syringe filled with cold complete culture medium containing 20% FCS. Cells were washed, plated at a density of approximately 2x106 cells/ml and treated with 20 ng/ml M-CSF for 6 days, with a medium change at day 3. Adherent cells were then harvested using a cell scraper and plated in complete medium containing 20% FCS and lacking M-CSF for 16 h. Cells were incubated the next day for 5 h in DMEM/1% FCS before staining for flow cytometry.
Instrument	Accuri C6 (BD BioScience)
Software	Accuri C6 software version 1.0-264.21 (BD BioScience)
Cell population abundance	MEF and THP1 samples were pure in the sense that no other cell types were present in the populations analyzed by flow

	cytometry. BMDMs were differentiated in vitro from crude bone marrow cultures. Non-adherent cells and debris were eliminated in 2-3 washing steps performed along the differentiation process. By the time differentiation was finished, the culture appeared rather clean, and the purity of the resulting macrophage population was establised by flow cytometry using anti-CD11b and anti-F4/80 markers (see Supplementary Fig. 16a)
Gating strategy	Cells were gated inthe FS/SS window to exclude dead cells and debris. The percentage of events selected in this window for further analysis of surface expression was always 80-90% for MEFs and THP1 cells, and aroung 70% for BMDMs. The gating strategies to define positive and negative populations are described in the respective figure legends, and a scheme for each strategy is provided in Supplementary Figure 17.

☒ Tick this box to confirm that a figure exemplifying the gating strategy is provided in the Supplementary Information.
